# Heterogeneous Acoustofluidic Distributions Induced by Different Radiation Surface Arrangements in Various Pseudo-Sierpiński-Carpet-Shaped Chambers

**DOI:** 10.3390/mi17020259

**Published:** 2026-02-16

**Authors:** Qiang Tang, Boyang Li, Chen Li, Junjie Wang, Huiyu Huang, Yulong Hu, Kan Zhu, Hao Chen, Xu Wang, Songfei Su

**Affiliations:** 1 Jiangsu Key Laboratory of Advanced Manufacturing Technology, Faculty of Mechanical and Material Engineering, Huaiyin Institute of Technology, Huaian 223003, China; m18205233053@163.com (B.L.); lc924938166@163.com (C.L.); curry_2026@foxmail.com (J.W.); hyhuang@hyit.edu.cn (H.H.); 15150706701@163.com (Y.H.); zhukan860109@hyit.edu.cn (K.Z.); chenhao@hyit.edu.cn (H.C.); 2 College of Mechanical and Electronic Engineering, Nanjing Forestry University, Nanjing 210037, China; schuewang@njfu.edu.cn; 3 School of Mechanical Engineering, Nanjing Institute of Technology, Nanjing 211167, China; susongfeinh@163.com

**Keywords:** non-traditional acoustofluidic distribution, pseudo-Sierpiński-carpet-shaped chamber, radiation surface arrangement, ultrasonic micro/nano-scale manipulation technology

## Abstract

In this research, an innovative scheme to generate heterogeneous acoustofluidic distributions in various pseudo-Sierpiński-carpet-shaped chambers with different filling fractions and cross-sectional configurations has been proposed and calculated for topographical manipulation of large-scale micro-particles. All of the structural components positioned in the pseudo-fractal chambers are symmetrically distributed in space, and all ultrasonic radiation surfaces hold the unified settings of input frequency point, oscillation amplitude, and initial phase distribution along their respective normal directions. A large number of fascinating acoustofluidic patterns can be generated in the originally-static pseudo-Sierpiński-carpet-shaped chambers at different recursion levels without complicated vibration parameter modulation. The simulation results of acoustofluidic distributions and particle motion trajectories under different radiation surface arrangements further demonstrate the manipulation performance of these specially designed devices, and indicate that controllable spatial partitioning and intensity modulation of the acoustofluidic field can be achieved by adjusting the hierarchical order, cross-sectional configuration and combination mode of the radiation surfaces. Unlike the existing device construction method of miniaturized microfluidic systems, the artificial introduction of fractal elements like Sierpiński carpet/triangle, Koch snowflake, Mandelbrot set, Pythagoras tree, etc., can provide extraordinary perspectives and expand the application range of the acoustofluidic effect, which also makes ultrasonic micro/nano-scale manipulation technology more abundant and diversified. This exploratory research indicates the potential possibility of applying fractal structures as alternative component parts to purposefully customize acoustofluidic distributions for the further research of patterned manipulation of bio-organisms and navigation of micro-robot swarms in brand new ways that cannot be achieved through traditional methods.

## 1. Introduction

Ultrasonic waves are mechanical longitudinal waves that propagate through elastic media and are typically generated using piezoelectric materials [1]. Their scientific origin can be traced to Carl von Linde’s pioneering research on underwater acoustics in 1883 [2,3]. After more than a century of development, ultrasound has combined the advantages of multiple disciplines and has become a transformative discipline with interdisciplinary impact. In the medical domain, by virtue of its non-ionizing radiation characteristic, real-time imaging capability, and cost-effectiveness, ultrasonic diagnostic technology has become an indispensable clinical tool [4,5,6]. It is widely used in disease monitoring and sample testing in obstetrics and gynecology, cardiovascular, digestive, and urinary systems [7,8,9,10]. Within the industrial sector, the transient high-temperature and high-pressure conditions induced by the acoustic cavitation effect significantly accelerate chemical reaction rates [11]. This mechanism is efficiently leveraged in precision cleaning, electrochemical synthesis enhancement, and advanced material processing [12,13,14]. In the biological sciences, micro-particle manipulation techniques based on acoustic radiation forces or acoustic streaming effects provide new paradigms for microfluidic chip design and high-sensitivity biosensing [15,16]. Although ultrasound has achieved significant advancements across numerous fields, the customized spatiotemporal modulation of complex acoustic fields remains a critical bottleneck impeding technological innovation. Customized spatiotemporal modulation fundamentally transforms acoustic manipulation technology from a static and homogeneous tool into a dynamic, intelligent, and highly adaptable physical platform. This is achieved through independent and precise programmed control over the phase, amplitude, and frequency of acoustic waves across both temporal and spatial dimensions [17]. By overcoming the limitations in flexibility and resolution inherent to traditional fixed acoustic fields, this technology enables high-throughput, high-precision, and low-damage parallel manipulation of cells, tissue engineering scaffolds, and drug carriers [18]. However, this technical approach still exhibits certain limitations. The precise spatiotemporal control of the acoustic field requires decoupling and independent control of the phase, amplitude, and frequency parameters of sound waves [19]. The nonlinear propagation, scattering, and attenuation effects of sound waves in complex media and multi-scale structures make precise modeling and real-time feedback control extremely difficult. At the level of device design and manufacturing, acoustic metasurfaces or transducer arrays that can achieve dynamic control require extremely high processing accuracy and integration density. Current micro/nano processing technology still remains inadequate in terms of high-performance piezoelectric material integration, three-dimensional heterostructure manufacturing, and driver circuit miniaturization [20]. Consequently, introducing fractal theory from nonlinear science to transcend the limitations of conventional modulation strategies will pioneer new pathways for the precise construction of patterned acoustic pressure fields and acoustic streaming vortices [21]. This advancement, in turn, is poised to drive innovation in cross-scale acoustic manipulation technology.

In recent years, the introduction of fractal structures into the design of acoustic metamaterials/metasurfaces, as replacements for conventional periodic structures, has emerged as a frontier research orientation in this research field [22,23,24]. The inherent self-similarity and non-integer dimensionality (Hausdorff dimension) of fractal geometry constitute key physical mechanisms for manipulating acoustic fields, particularly ultrasonic fields [25,26]. The multi-scale recursively nested geometric configuration of fractals enables microcavities or resonant units of distinct characteristic scales to couple with specific frequency bands, thereby generating discrete multi-band responses in the oscillation frequency spectrum [27,28,29]. This scale invariance is intrinsically linked to the Hausdorff dimension, and as the dimension increases, heightened topological complexity induces a spatial folding effect on acoustic wave propagation paths [30,31]. This effect significantly extends the effective acoustic path length within a confined volume. Simultaneously, by enhancing multiple scattering of sound waves, fractal structures can effectively induce strong acoustic localization effects. The substantial extension of the effective acoustic path length achieved by high-dimensional fractal structures markedly enhances sound absorption and insulation performance. Furthermore, the resulting strong acoustic localization effects not only intensify micro-vortical acoustic streaming, providing novel pathways for the efficient capture of micro/nano particles and rapid fluid mixing, but also lay the physical groundwork for realizing highly integrated acoustofluidic chips [32,33], high-sensitivity acoustic sensing platforms, and multifunctional acoustic manipulation systems [34,35,36,37]. This demonstrates the immense potential of fractal-based designs in the development of cross-scale acoustic devices.

In the field of micro/nano-scale manipulation, traditional uniform acoustic field systems are insufficient to achieve complex, heterogeneous, and programmable spatial arrangement of particles or biological samples, which limits the potential of acoustic technology in cutting-edge applications such as microrobot collaborative motion, multicellular tissue construction, high-throughput biological detection, and so on [38]. The innovative acoustofluidic generation strategy based on the pseudo-Sierpiński-carpet-shaped chamber proposed in this work can effectively address this core issue. By introducing fractal-inspired multi-scale geometric structures and independently controllable radiation surface arrays, it provides a new approach for spatial customization of acoustic energy distribution and acoustic streaming fields. This scheme can generate diverse acoustofluidic patterns by adjusting the geometric arrangement and the radiation surface combination under single frequency point excitation, oscillation amplitude, and initial phase distribution. In comparison with the strictly defined fractal geometry, the introduction of pseudo-fractal structures has the physical advantage of being composed of regularized multi-stage features like circular or polygonal cross-sectional elements, avoiding the limitation of rigorous fractal self-similarity on sound field distribution, allowing the acoustic streaming vortices to present diversified patterns according to the combination of different radiation surfaces, and expanding the design redundancy for acoustofluidic regulation. This structural construction strategy is suitable for microfluidic and acoustic manipulation scenarios that require diverse acoustofluidic modes and focus on functionality, such as flexible capture, efficient sorting, and precise transport of biological particles, multi-phase mixing, and directional navigation of micro/nano-robots [39]. Our exploratory research indicates the possibility of utilizing fractal elements as alternative components to customize acoustofluidic distributions for the further investigation of next-generation acoustic microrobots, morphological construction of biological tissues, and integrated multifunctional acoustic micro-control systems in ways which cannot be achieved via traditional methods, which provides key technical ideas and design platforms for surpassing traditional uniform sound field limitations and achieving cross-scale programmable acoustofluidic manipulation.

## 2. Theory and Methods

### 2.1. The Perturbation Theory

The generation principium and governing equations of acoustofluidic fields have been elucidated by other research groups [40,41]. In this paper, perturbation theory is briefly introduced to provide an instructive description for the following calculation process of acoustofluidics. The mass and momentum conservation equations describing the homogeneous and isotropic Newtonian fluid motion can be expressed as follows:

(1) $$\frac{\partial \rho }{\partial t}+\nabla \cdot (\rho u)=0,$$

(2) $$\rho (\frac{\partial u}{\partial t}+u\cdot \nabla u)=-\nabla p+\mu \nabla^{2}u+(\mu_{b}+\frac{1}{3}\mu )\nabla \nabla \cdot u,$$

where *ρ*, *μ* and *μ_b_* are respectively the density, shear and bulk viscosity coefficients of the given fluid medium; *p* and ***u*** represent the disturbed pressure field and Eulerian velocity vector originating from the fluidic movement, respectively. The left side of Equation (2) represents the unit volume inertial force acting on the Newtonian fluid medium, and the two terms in the parenthesis denote the local acceleration and convective acceleration, respectively, while the terms on the right side of Equation (2) include the stress variations such as the pressure gradient and the viscous forces. Other forces, like gravity force and buoyancy force, are not taken into consideration as they are almost negligible in comparison with the aforementioned driving forces [42].

Under the premise of slight oscillation, the physical parameters of the given fluid medium including density *ρ*, pressure *p*, and velocity vector ***u*** can be respectively expanded into the following forms.

(3) $$\rho =\rho_{0}+\rho_{1}+\rho_{2}+\cdots ,$$

(4) $$p=p_{0}+p_{1}+p_{2}+\cdots ,$$

(5) $$u=u_{1}+u_{2}+\cdots ,$$

where the subscript symbols 0, 1, and 2 respectively represent the steady-state (undisturbed fluid medium), first-order (ultrasonic oscillation), and second-order (acoustic streaming) components. The higher-order terms represented by ellipsis dots in Equations (3)–(5) can be neglected because they belong to infinitesimal quantities in the following calculation process. By substituting Equations (3)–(5) into Equations (1) and (2), the following first-order sound field expression can be obtained.

(6) $$\frac{\partial \rho_{1}}{\partial t}+\rho_{0}\nabla \cdot u_{1}=0,$$

(7) $$\rho_{0}\frac{\partial u_{1}}{\partial t}=-\nabla p_{1}+\mu \nabla^{2}u_{1}+(\mu_{b}+\frac{1}{3}\mu )\nabla \nabla \cdot u_{1}.$$

Repeating the above-mentioned operation procedure and considering time-varying cycle average within one or more oscillation periods, the second-order acoustic streaming field can also be written as follows:

(8) $$\bar{\frac{\partial \rho_{2}}{\partial t}}+\rho_{0}\nabla \cdot \bar{u_{2}}+\nabla \cdot \bar{\rho_{1}u_{1}}=0,$$

(9) $$\rho_{0}\bar{\frac{\partial u_{2}}{\partial t}}+\bar{\rho_{1}\frac{\partial u_{1}}{\partial t}}+\rho_{0}\bar{u_{1}\cdot \nabla u_{1}}=-\nabla \bar{p_{2}}+\mu \nabla^{2}\bar{u_{2}}+(\mu_{b}+\frac{1}{3}\mu )\nabla \nabla \cdot \bar{u_{2}}.$$

The overbar operative notation $\bar{\phi (r,t)}=\frac{1}{T}\int_{T}\phi (r,t)dt$ represents time average value of a given physical field variable within one vibration period *T*. Due to the fact that the second-order acoustic streaming field is steady-state and independent of time *t*, $\bar{\frac{\partial \rho_{2}}{\partial t}}=0$, $\bar{\frac{\partial u_{2}}{\partial t}}=0$, $\bar{u_{2}}=u_{2}$, and $\bar{p_{2}}=p_{2}$. Therefore, Equations (8) and (9) can be eventually simplified as the following:

(10) $$\rho_{0}\nabla \cdot u_{2}=-\nabla \cdot \bar{\rho_{1}u_{1}},$$

(11) $$\nabla p_{2}-\mu \nabla^{2}u_{2}-(\mu_{b}+\frac{1}{3}\mu )\nabla \nabla \cdot u_{2}=-(\rho_{0}\bar{u_{1}\cdot \nabla u_{1}}+\bar{\rho_{1}\frac{\partial u_{1}}{\partial t}}).$$

where ***u***_2_ and *p*_2_ respectively represent the time-independent flow velocity vector and the pressure distribution located in the acoustic streaming field.

### 2.2. The Simulation Method

Utilizing the commercial finite element analysis software COMSOL Multiphysics (version 5.5, COMSOL AB, Stockholm, Sweden), the entire simulation process of acoustofluidics can be decomposed into three elementary steps [43].

In the first step, by combining Equations (6) and (7) with appropriate acoustic boundaries, the ‘Thermoviscous Acoustics, Frequency Domain’ module can be applied to simulate the sound pressure distribution and oscillation velocity field generated by different radiation surface arrangements. The initial values and acoustic boundary conditions are set as follows: the vibration amplitude, input frequency point and initial phase of all radiation surfaces are user-defined, while the remaining fluid–solid interfaces are uniformly set as isothermal and non-slip.

In the second step, on the basis of the sound pressure distribution and vibration velocity field computed under the given oscillation conditions, variable predefined operations in COMSOL Multiphysics can be applied to calculate the mass source term $-\nabla \cdot \bar{\rho_{1}u_{1}}$ in Equation (10) and the volume force term $-(\rho_{0}\bar{u_{1}\cdot \nabla u_{1}}+\bar{\rho_{1}\frac{\partial u_{1}}{\partial t}})$ in Equation (11), both of which originate from the non-uniformity characteristics of ultrasonic physical quantities in the spatiotemporal distribution and act as the driving forces for steady-state acoustic streaming.

In the third step, based on Equations (10) and (11), the fluid dynamics module ‘Laminar Flow’ can be utilized to simulate the second-order acoustic streaming field. In comparison with the mass source and volume force terms under the circumstance of low Reynolds number, the inertial force term $\rho_{0}(u_{2}\cdot \nabla )u_{2}$ can be artificially neglected in the following simulation of time-independent acoustic streaming distribution. All fluidic boundaries are uniformly set to be non-slip. Additionally, in order to ensure the calculation convergence of flow field, the weak contributions of mass source term and acoustic streaming pressure are indispensable and need to be taken into account.

On the basis of the computed acoustofluidic field, the ‘Particle Tracing for Fluid’ module can also be used to simulate the particle movement trajectory under the coaction of acoustic radiation force ***F****_rad_* (i.e., acoustophoretic force) and Stokesian drag force ***F****_drag_* induced by the acoustic streaming field.

(12) $$F_{rad}=-\frac{4}{3}\pi R_{p}^{3}\nabla (\frac{1-\beta }{2\rho_{0}c_{0}^{2}}\bar{p_{1}^{2}}-\frac{D}{2}\rho_{0}\bar{{\left\|u_{1}\right\|}^{2}}),$$

(13) $$F_{drag}=6\pi \mu R_{p}(u_{2}-u_{p}).$$

where *R_p_* and ***u****_p_* represent the radius and kinematic velocity of manipulated particles, respectively. The parameters *β* and *D* are defined as follows:

(14) $$\beta =\frac{\rho_{0}c_{0}^{2}}{\rho_{p}c_{p}^{2}},$$

(15) $$D=\frac{3(\rho_{p}-\rho_{0})}{2\rho_{p}+\rho_{0}}.$$

where *ρ_p_* and *c_p_* refer to the particle’s density and sound speed, respectively. In the simulation process of particle movement trajectory, Newton’s second law of motion is taken into consideration, ignoring the buoyancy force and gravity force acting on micro-scale particles suspended in the fluid medium.

(16) $$\frac{d}{dt}(\rho_{p}\frac{4}{3}\pi R_{p}^{3}u_{p})=F_{rad}+F_{drag}.$$

### 2.3. Brief Introduction to Fractal Theory

Fractal theory is acclaimed as a geometric framework for describing complex natural morphology. Although it falls within the discipline of modern mathematics, its essence transcends the scope of traditional geometry, offering a novel worldview and methodology. The fractal dimension, as a central concept of this theory, quantitatively characterizes the efficiency with which a complex structure fills its embedded space, while also serving as a precise measure of its irregularity or discontinuity. For a strictly self-similar fractal structure, its Hausdorff dimension (i.e., self-similarity dimension) *D* can be precisely defined by the following formula [25,26]:

(17) D=ln(M)/ln(N).

According to the above-mentioned definition, the fractal dimension *D* is typically a non-integer value. *M* represents the number of subsets that are self-similar to the whole structure after the decomposition of the fractal. *N* is the scaling factor, referring to the ratio by which each self-similar subset is reduced relative to the original structure along the linear scale. Although the pseudo-Sierpiński carpet introduced in this work is not constructed in strict accordance with self-similarity principles, its dimension can still be estimated using the aforementioned dimensional formula, yielding an approximate value of 1.8928. By analyzing the distribution characteristics of the ultrasonic field presented in the following section, a clear and distinct correlation emerges between the acoustofluidic patterns and the concepts of self-similarity and fractal dimension.

Subsequent calculations reveal that fractal structures serving as acoustofluidic boundaries introduce highly complicated geometric morphology constraints. When solving the governing equations for the first-order acoustic field, such boundaries disrupt conventional regularity, leading to solutions that exhibit multi-scale, quasi-periodic, or localized characteristics in space, thereby laying the foundation for constructing heterogeneous acoustofluidic fields. By modulating the distributions of acoustic pressure and fluid velocity, fractal structures cause the quadratic terms of acoustic pressure and flow velocity to form spatially non-uniform distributions. As a result, the acoustofluidics-induced driving force determined by gradient terms manifests as a complex yet predictable force-field configuration. Based on this mechanism, selective particle capture, precise sorting, and transport along complex trajectories can be achieved using only single-frequency excitation. Furthermore, fractal structures significantly enhance flow-field control capabilities through their influence on acoustic streaming mechanisms. Their geometric morphology incorporates numerous multi-scale sharp edges and corners. In regions near these features, the first-order acoustic velocity field undergoes drastic changes, inducing substantial velocity gradients. These gradients, in turn, lead to localized enhancement of Reynolds stress, forming high-strength and spatially complex acoustic streaming source terms. This ultimately drives the generation of a mean flow field characterized by multi-vortex structures and hierarchical features. Such complex acoustic streaming fields arising from fractal boundaries can greatly enhance fluid mixing efficiency in microchannels, effectively overcoming diffusion limitations, and provide a versatile hydrodynamic environment for micro/nano-scale particle manipulation.

## 3. Results and Discussion

**Figure 1:**
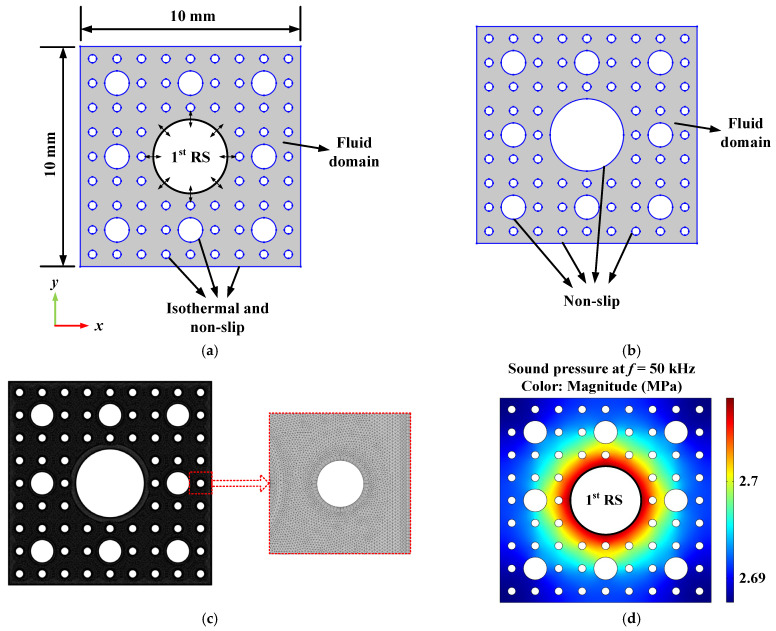

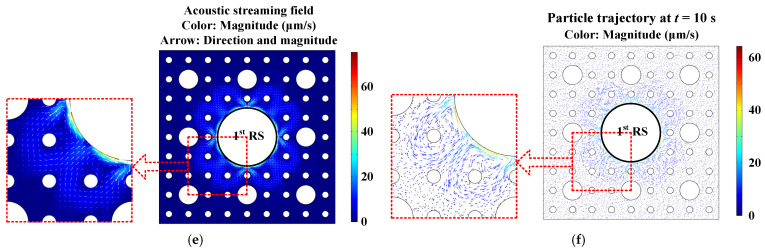
(Color online). Acoustofluidic field and particle trajectory generated in the 3-stage pseudo-Sierpiński-carpet-shaped chamber with circular cross-section under the excitation of 1st RS. (**a**) Computational model and boundary condition of sound field. (**b**) Computational model and boundary condition of acoustic streaming field. (**c**) Meshed model. (**d**) Pattern of sound pressure field. (**e**) Pattern of acoustic streaming field. (**f**) Pattern of micro particle trajectory at a given time (10 s).

In order to validate the aforementioned simulation method and process, an original scheme to generate heterogeneous acoustofluidic distributions and particle movement trajectories caused by assorted radiation surface arrangements in different pseudo-Sierpiński-carpet-shaped chambers with various filling fractions and cross-sectional configurations is put forward. According to the existing references [44,45], when the height/thickness of the microfluidic chamber can be ignored in comparison with the other two dimensions, the original 3D (three-dimensional) bulk-wave-driven acoustic streaming field can be simplified into a 2D (two-dimensional) model. Although there are differences in the magnitude of acoustic streaming velocity, the acoustofluidic patterns at different heights of the horizontal plane are basically similar, and the flow speeds can be easily regulated by changing the input voltage values of piezoelectric transducers. Therefore, although it is necessary to consider the pseudo-Sierpiński-carpet-shaped chambers as bulk wave devices in practice, simplified 2D acoustofluidic models can still be used in the following simulation process, and the possible manufacturing method and excitation mode of the 3D pseudo-Sierpiński-carpet-shaped chambers are depicted in Figure S1 (Schematic diagram of fabrication method and oscillation mode) of the Supplementary Materials. The 3D pseudo-Sierpiński-carpet-shaped chambers with different filling fractions and cross-sectional configurations can be constructed using traditional soft lithography or 3D microprinting technique. As described elsewhere in the literature, thin film ultrasonic transducers can be attached to the upper surface of each fractal convex platform [39,46]. As shown in Figure S1, the vertical oscillation of each thin film ultrasonic transducer can be further transformed into the normal vibration of the corresponding radiation surface through the Poisson effect. Geometrically speaking, the 2D pseudo-Sierpiński-carpet-shaped chambers with different filling fractions and cross-sectional configurations in our simulation can be feasibly constructed using the following procedure [47,48,49]. For example, a square chamber with a side length of *L*_0_ = 10 mm is divided into 9 congruent sub-square regions in a 3 × 3 grid. By removing a circle with a diameter (10/3 mm) equal to the side length of the central sub-square region, a 1-stage pseudo-Sierpiński-carpet-shaped chamber with circular cross-section can be created. Similarly, the same step can be repeated in the eight remaining regions to generate a 2-stage pseudo-Sierpiński-carpet-shaped chamber with circular cross-section. Although the above-mentioned process can continue indefinitely, considering the construction limitations of the device-manufacturing equipment and the computational performance of our workstation, the subdivision operation must be terminated at a specific step *n*, and an *n*-stage pseudo-Sierpiński-carpet-shaped chamber with circular cross-section can be ultimately generated. In the following simulation, a 3-stage pseudo-Sierpiński-carpet-shaped chamber with circular cross-section is primarily used to calculate the sound field and acoustic streaming distribution with the given boundary conditions, as shown in Figure 1a,b, respectively. The mesh refinement scheme with locally magnified boundary layer setting is illustrated in Figure 1c. Most simulation regions are divided into free triangular grids with a maximum size of 0.1 mm, which is 1/300 of the sound wavelength at 50 kHz ($\lambda =\frac{c_{0}}{f}=\frac{1500\;m/s}{50\;\mathrm{kHz}}=30\;\mathrm{mm}$). The boundary layer number and the first element thickness are respectively defined as 6 and 0.5 μm, which is 1/5 of the flow boundary layer thickness in water ($\delta =\sqrt{\frac{\mu }{\pi \rho_{0}f}}=\sqrt{\frac{0.001\;\mathrm{Pa}\cdot s}{\pi \times 1000{\;\mathrm{kg}/m}^{3}\times 50\;\mathrm{kHz}}}\approx 2.5\;\mu m$) when the oscillation frequency is 50 kHz. Unless otherwise specified, the model parameters throughout the entire simulation process, including structural dimensions, material properties and operating conditions, are listed in Table 1 (Model parameters in the simulation) as far as possible.

### Model Validation

**Figure 2:**
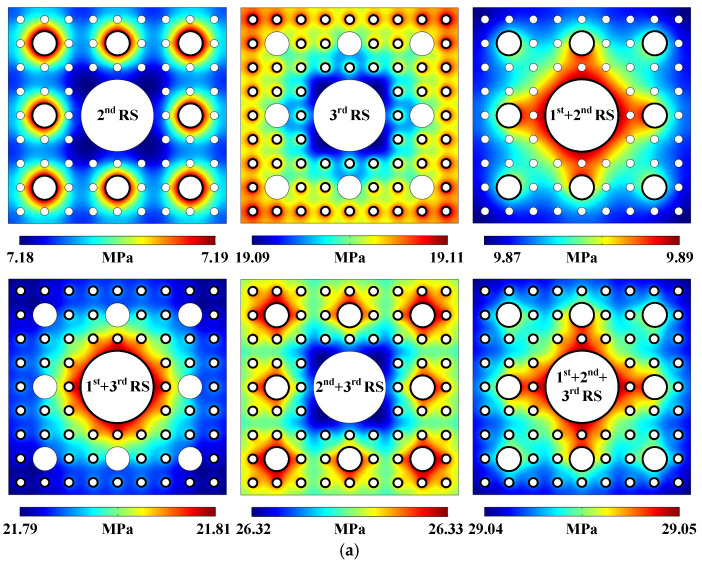

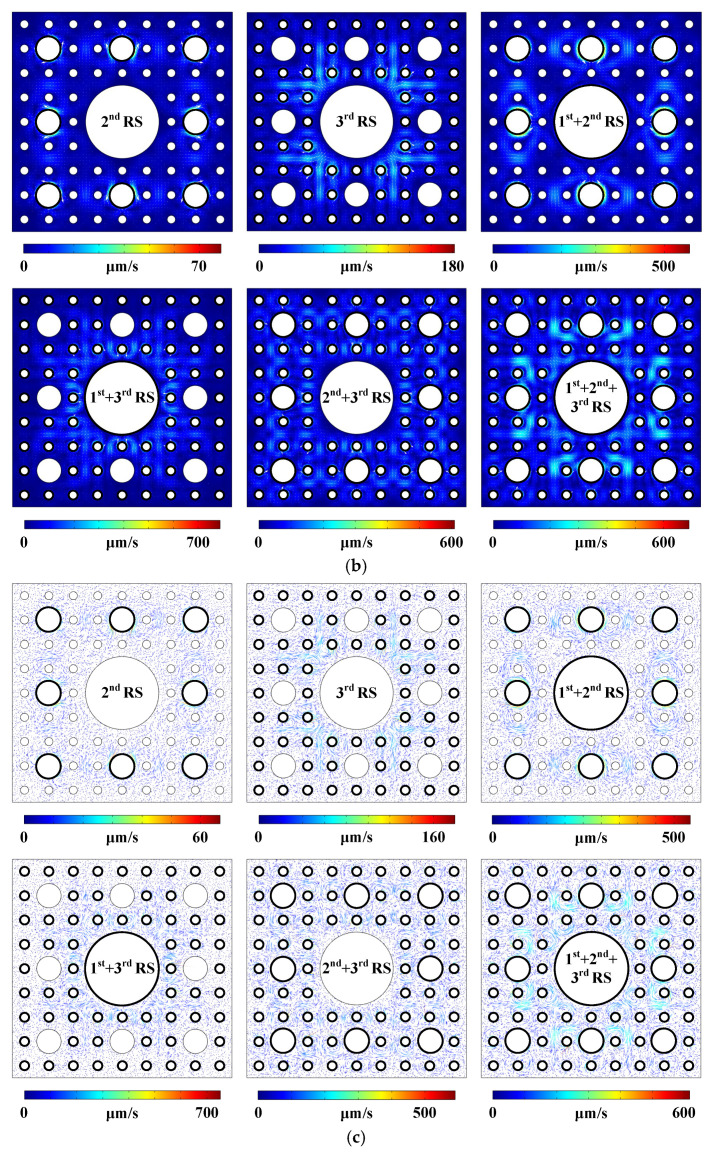

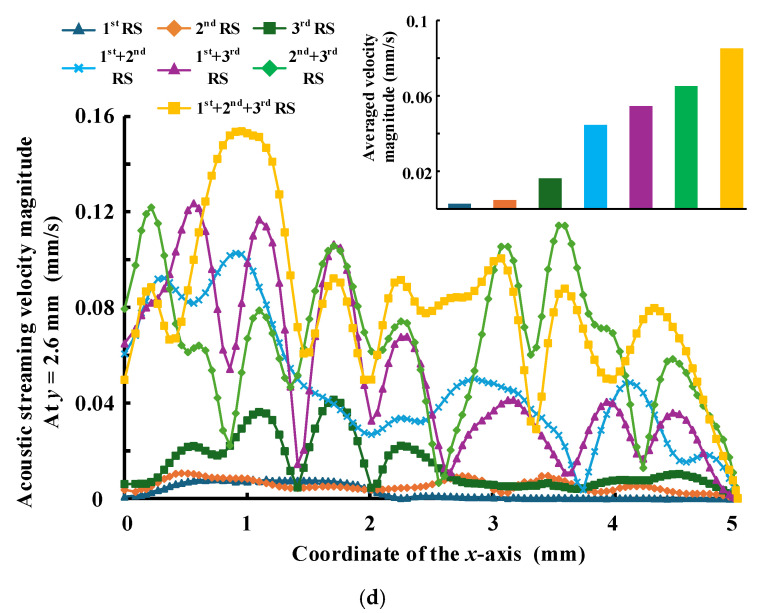
(Color online). Acoustofluidic fields and particle trajectories generated in the 3-stage pseudo-Sierpiński-carpet-shaped chamber with circular cross-section under the excitation of different-stage radiation surfaces. (**a**) Pattern of sound pressure field. (**b**) Pattern of acoustic streaming field. (**c**) Pattern of micro particle trajectory at a given time (10 s). (**d**) Acoustic streaming velocity magnitude distribution.

The simulated sound pressure distribution and acoustic streaming field generated in the 3-stage pseudo-Sierpiński-carpet-shaped chamber with circular cross-section under the excitation of 1-stage radiation surfaces (abbreviated as 1st RS) highlighted by the black solid lines are simulated and respectively plotted in Figure 1d (Pattern of sound pressure field) and Figure 1e (Pattern of acoustic streaming field). All radiation surfaces possess the same input frequency point of 50 kHz, vibration amplitude of 100 nm, and initial phase distribution along normal directions, as indicated by double-headed arrows in Figure 1a (Computational model and boundary condition of sound field). Due to the presence of higher-stage sub-circular regions, ultrasound will reflect and diffract among multiple fluid–solid interfaces, resulting in a symmetrical sound field mode with discontinuous and uneven boundaries, but overall still exhibiting a concentric circular distribution. However, unlike the global characteristic of sound pressure distribution, the petal-shaped acoustic streaming field is roughly limited to the vicinity of 1-stage radiation surfaces and the adjacent fluid–solid boundaries, and mainly flows out from the four directions of 0°, 90°, 180°, and 270°, whilst flowing in from the other four directions of 45°, 135°, 225°, and 315° in the Cartesian coordinate system, as shown in the enlarged red dashed box in Figure 1e. The above-mentioned conclusion can also be verified by the movement trajectory pattern of massive particles, which basically rotate along the eight local vortices symmetrically distributed around the 1-stage radiation surfaces, as shown in Figure 1f (Pattern of micro particle trajectory at a given time). According to our previously published paper [50] and the comparison among the magnitudes of acoustic radiation force, acoustic streaming induced drag force, gravity force, and buoyancy force in the Supplementary Material, the buoyancy force and gravity force can be neglected compared to the acoustic radiation force and the Stokesian drag force acting on polystyrene beads with a diameter of 1 μm, as shown in Figure S2 (Driving force magnitudes generated in the 3-stage pseudo-Sierpiński-carpet-shaped chamber with circular cross-section under the excitation of 1st RS). In addition, the distribution of acoustic radiation force magnitude within the whole chamber is relatively uniform and almost concentrated near the radiation surfaces. Therefore, the particle motion pattern in the proposed pseudo-Sierpiński-carpet-shaped chamber is generally consistent with the distribution of the acoustic streaming field. The particle movement trajectory patterns in the following simulation results also meet the above conclusion. In addition to Figure 1 (Acoustofluidic field and particle trajectory generated in the 3-stage pseudo-Sierpiński-carpet-shaped chamber with circular cross-section under the excitation of 1st RS), the remaining combination modes and the corresponding acoustofluidic distributions of 1- to 3-stage radiation surfaces, as well as particle motion trajectories, are plotted in Figure 2 (Acoustofluidic fields and particle trajectories generated in the 3-stage pseudo-Sierpiński-carpet-shaped chamber with circular cross-section under the excitation of different-stage radiation surfaces) without omission, thus demonstrating the influence of different vibration source arrangements on the acoustic streaming fields. Since all initial phase settings remain unchanged in all simulations, the combination of multi-stage radiation surfaces can be directly abbreviated as *m*th + *n*th + ⋯ RS, such as 1st + 2nd + 3rd RS in Figure 2a (Pattern of sound pressure field).

In comparison with the sound field generated by the vibration of the 1-stage radiation surfaces, the sound pressure distribution excited by the 2-stage radiation surfaces possesses the self-similarity characteristic of the fractal structure, and the regions with relatively higher sound pressure values are almost concentrated near the corresponding vibration sources. This is due to the fact that when the lower-stage radiation surfaces (1- or 2-stage) oscillate, the higher-stage radiation surfaces in the orientations of 45°, 135°, 225°, and 315° are farther away from the lower-stage radiation surfaces, making it hard for them to affect the sound pressure distribution generated by the lower-stage radiation surfaces. The distribution of sound pressure generated by the 3-stage radiation surfaces is different from that induced by the 1- or 2-stage radiation surfaces, and the areas with higher sound pressure values are almost concentrated around the peripheries of the 3-stage pseudo-Sierpiński-carpet-shaped chamber, especially at the four corners. This occurs because the chamber boundaries are closer to the 3-stage radiation surfaces, and the local sound pressure distribution at the four corners is mainly affected by the corresponding two boundaries of the chamber. From Figure 1d and Figure 2a, it can be seen that the sound pressure magnitudes produced by the fluctuation of *m*th + *n*th + ⋯ RS satisfy the linear superposition of those induced by individual radiation surfaces, while the sound pressure patterns do not follow the above principle. Comparing the circumstances of the sound pressure distributions generated by 1st RS, 1st + 2nd RS, 1st + 3rd RS, and 1st + 2nd + 3rd RS, it can be concluded that the overall sound pressure distribution is primarily determined by the lowest-stage radiation surfaces, and the existence of higher-stage radiation surfaces only affects the local sound field. The higher-stage radiation surfaces located along the diagonal directions basically have no influence on the sound pressure patterns, which is due to the fact that they are farther away from the lowest ones, and the regions with larger sound pressure values are predominantly concentrated in the area surrounding the 1st RS. Similarly, the sound pressure distributions of 2nd RS and 2nd + 3rd RS also follow the aforementioned principle. Overall, the sound fields involved in the 3-stage pseudo-Sierpiński-carpet-shaped chamber do not completely follow the self-similarity feature of the fractal structure, which can be obtained by comparing the sound pressure distribution generated by the 3-stage radiation surfaces with the remaining situations. The above-mentioned conclusion is quite different from that in our previous published paper [39], which is due to the fact that the construction process of the pseudo-Sierpiński-carpet-shaped chamber does not strictly follow the fractal principle.

Comparing Figure 1e and Figure 2b (Pattern of acoustic streaming field), from the perspective of acoustic streaming distribution, the influence range of radiation surfaces is no longer determined by the lowest-stage radiation surfaces, and the acoustic streaming field caused by individual higher-stage radiation surfaces cannot be directly obtained by scaling the field generated by the lower-stage radiation surfaces. The influence of the higher-stage radiation surfaces on the acoustic streaming field is greater than that of the low-stage radiation surfaces, to a certain extent. Once the highest-stage (3-stage) radiation surfaces participate in oscillation, strip-shaped acoustic streaming distribution will be formed, which is due to the fact that the highest-stage radiation surfaces possess the widest distribution area and the largest influence range, and the distance between opposite radiation surfaces is the closest. Therefore, diffraction and reflection effects during sound wave propagation are more likely to occur, and acoustic energy is more concentrated in given areas, which is capable of driving acoustic streaming over a larger scale. Under the combination circumstance of multi-stage radiation surfaces, the flow orientations of the acoustic streaming field induced by the oscillation of the higher-stage fluid–solid interfaces point towards the secondary- or lowest-stage radiation surfaces from a global perspective. Meanwhile, the local acoustic stream flowing out from the higher-stage radiation surfaces will preferentially point towards the neighboring lower-stage solid–liquid interfaces. The particle trajectory diagram in Figure 2c (Pattern of micro particle trajectory at a given time) also illustrates that polystyrene beads are basically confined to the near-field region of vibration sources under the influence of the lower-stage radiation surfaces. However, in the presence of the highest-stage vibration sources, the movement range of massive polystyrene particles will inevitably extend to a wider area within the chamber.

In order to quantitatively depict the distribution of an acoustic streaming field excited by different combination modes of multiple radiation surfaces, the acoustic streaming velocity magnitude $\left\|u_{2}\right\|$ along the *x*-axis at *y* = 2.6 mm is extracted from Figure 1e and Figure 2b, and the obtained curves can be regarded as characteristic lines describing the acoustic streaming distributions, as shown in Figure 2d (Acoustic streaming velocity magnitude distribution). Although the number of extreme values in each curve and the corresponding *x*-axis coordinates are totally different from each other, the extreme value numbers in the characteristic lines acquired from the combination modes are obviously larger than those generated by individual radiation surfaces, which is due to the fact that under the influence of multiple vibration sources, more local small vortices can be generated. In addition, the column chart representing the averaged acoustic streaming velocity magnitudes $\bar{u_{2}}$ in the 3-stage pseudo-Sierpiński-carpet-shaped chamber under different radiation surface combination modes are also plotted in Figure 2d, which can be calculated by the following formula:

(18) $$\bar{u_{2}}=\frac{1}{S}\iint_{S}\left\|u_{2}\right\|dxdy,$$

where *S* denotes the *n*-stage pseudo-Sierpiński-carpet chamber area and can be expressed as $\frac{1}{4}L_{0}^{2}\left[4+\pi {\left(\frac{8}{9}\right)}^{n}-\pi \right]$. The different colors used in the column chart represent different radiation surface combination modes, which is consistent with the colors of the characteristic curves. From the perspective of energy conversion, the introduction of higher-stage radiation surfaces will transmit more sound energy into the fluid medium, which will result in larger fluid flow velocity magnitude and wider acoustic streaming range. Assuming that the height of the pseudo-Sierpiński-carpet-shaped chamber is *H* (see the Supplementary Material), the total area of the corresponding radiation surfaces of different stages can be expressed as $\sum_{n}\frac{8^{n-1}}{3^{n}}HL_{0}\pi$. Therefore, the area ratio of 1st RS, 2nd RS, 3rd RS, 1st + 3rd RS, 2nd + 3rd RS, and 1st + 2nd + 3rd RS can be written as 9:24:64:33:73:88:97, while the corresponding averaged acoustic streaming velocity magnitude is about 3:4:19:41:56:63:82. According to the column chart, it can be found that even though the averaged fluid flow speed under two single modes is small, the averaged acoustic streaming velocity magnitude after recombination will still be significantly enhanced. The reason is that the sound field between adjacent radiation surfaces is concentrated due to the superposition effect, resulting in an increase in the sound energy gradient within the entire chamber, and correspondingly an increase in acoustic streaming velocity magnitude.

To sum up, in the pseudo-Sierpiński-carpet-shaped chamber, the lower-stage radiation surfaces play a crucial role in sound pressure distribution. However, for the acoustic streaming field induced by the nonlinear effect of ultrasound, the existence of higher-stage vibration sources will affect the acoustofluidic pattern, which occurs because sound waves can bypass obstacles by diffraction, while acoustic streaming vortices will be hindered by these obstacles along the flow pathway. Also, in comparison with our previous work [39], due to the fact that the circular radiation surfaces do not contain sharp corners, it is difficult to form a concentrated strong sound field in the whole 3-stage pseudo-Sierpiński-carpet-shaped chamber, resulting in the inability to generate pronounced sound intensity gradients, and thus the range and magnitude of the acoustic streaming field will be correspondingly weakened.

**Figure 3:**
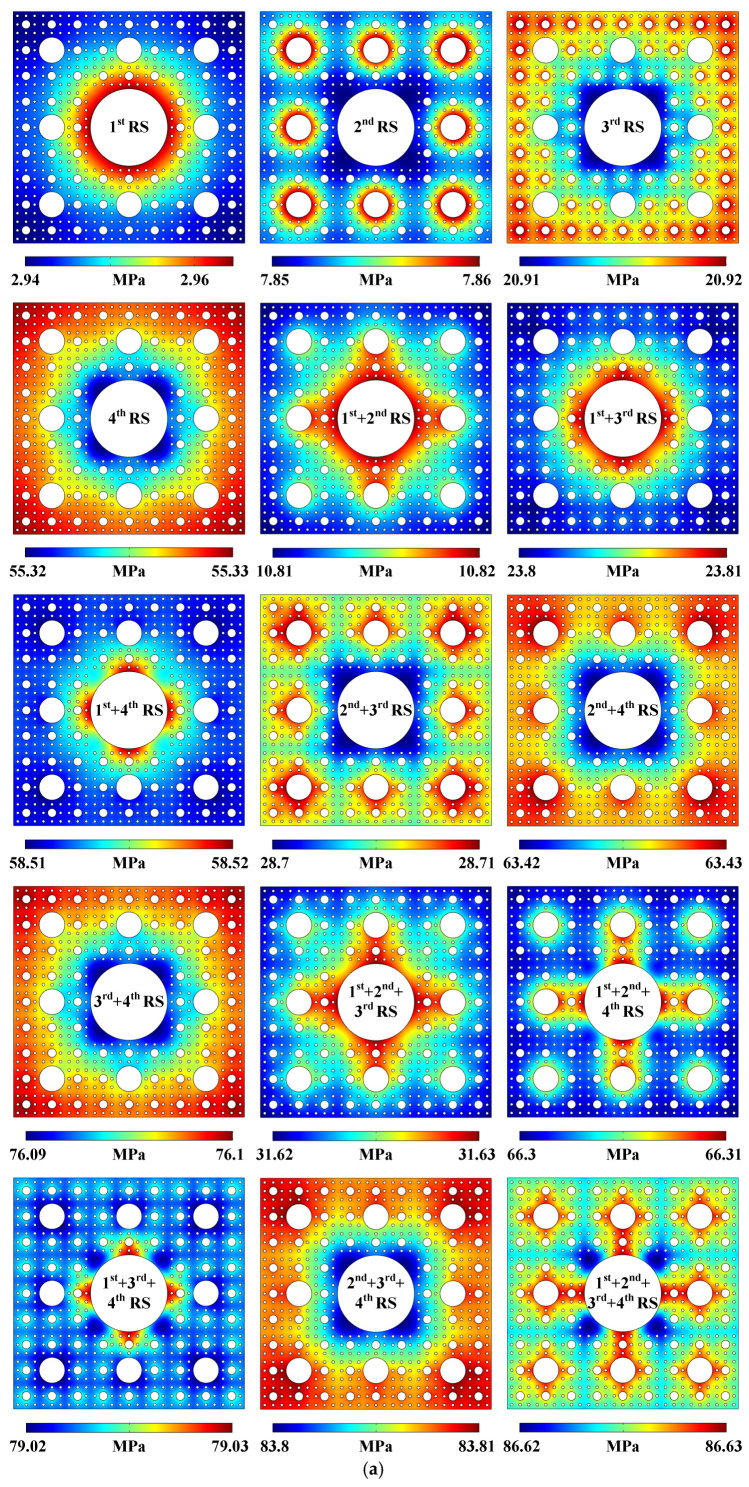

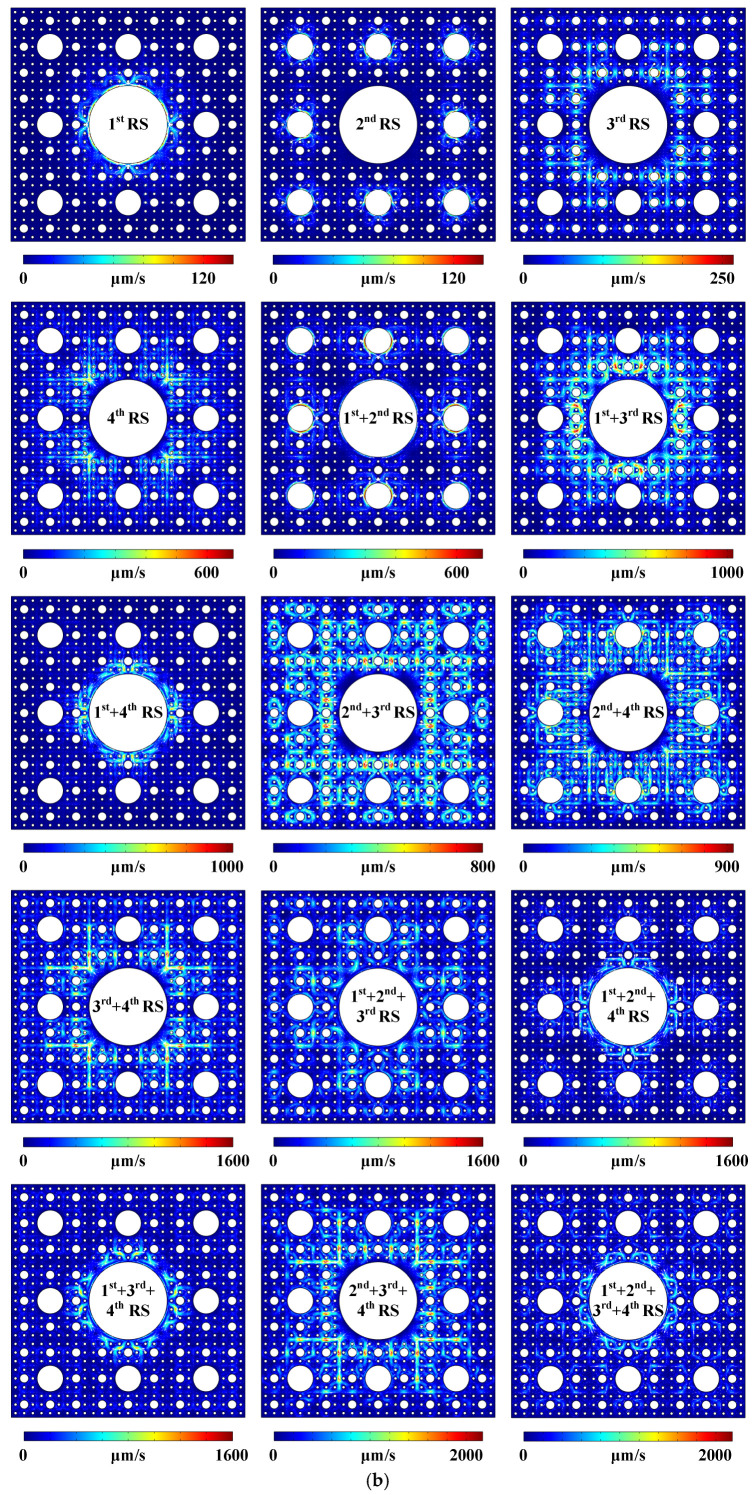

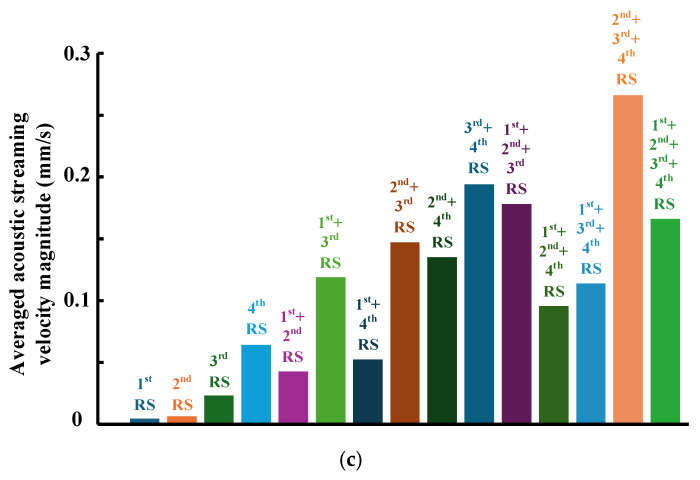
(Color online). Acoustofluidic fields and particle trajectories generated in the 4-stage pseudo-Sierpiński-carpet-shaped chamber with circular cross-section under the excitation of different-stage radiation surfaces. (**a**) Pattern of sound pressure field. (**b**) Pattern of acoustic streaming field. (**c**) Averaged acoustic streaming velocity magnitude.

More patterned sound pressure distributions, acoustic streaming fields, particle movement trajectories, and the averaged acoustic streaming velocity magnitudes generated in the 4-stage pseudo-Sierpiński-carpet-shaped chamber with circular cross-section under the excitation of different-stage radiation surface combination modes are all plotted in Figure 3 (Acoustofluidic fields and particle trajectories generated in the 4-stage pseudo-Sierpiński-carpet-shaped chamber with circular cross-section under the excitation of different-stage radiation surfaces). The evolution law of the sound field under the combination of 1- to 4-stage radiation surfaces is consistent with the above-mentioned conclusion drawn from Figure 1d and Figure 2a. When the lower-stage radiation surfaces (1st RS or 2nd RS) experience oscillation, the generated extreme sound pressure regions are almost concentrated near the corresponding vibration sources. However, in comparison with the sound pressure distributions generated in the 3-stage pseudo-Sierpiński-carpet-shaped chamber under the same radiation surface modes, the sound field contours shown in Figure 3a (Pattern of sound pressure field) tend to be more circular, which are determined by the denser arrangement of the 4-stage radiation surfaces. When the acoustic waves pass through the 4-stage fluid–solid interfaces, part of the sound energy along the directions of 0°, 90°, 180°, and 270° will be dissipated, thereby homogenizing the sound field distribution. Similarly, the regions of larger sound pressure generated by the vibration of higher-stage radiation surfaces (3rd RS or 4th RS) are still concentrated around the peripheries of the 4-stage pseudo-Sierpiński-carpet-shaped chamber, especially at the four corners, which occurs because the chamber boundaries are much closer to the higher-stage radiation surface, making it easier for the sound field to be reflected and form a standing wave distribution. Under the combination modes of multiple-stage vibration sources, the sound pressure distribution exhibits nonlinear superposition characteristic. In the presence of lower-stage vibration sources participating in oscillation, the spatial distribution of sound pressure is mainly determined by the lower-stage radiation surfaces, while the existence of higher-stage solid–liquid interfaces only affects the local sound pressure patterns. Similarly to the situation shown in Figure 1d and Figure 2a, the sound pressure values generated by different combination modes of multiple-stage radiation surfaces in the 4-stage pseudo-Sierpiński-carpet-shaped chamber still follow the linear superposition law of the sound pressure induced by single radiation surfaces.

Figure 3b (Pattern of acoustic streaming field) shows the acoustic streaming distributions under all possible combination modes of 1- to 4-stage radiation surfaces. It can be found that the introduction of higher-stage radiation surfaces usually leads to a strip-shaped fluid field. The reason is that the higher-stage radiation surfaces possess denser arrangement and wider distribution range, and when the lower-stage radiation surfaces are involved in vibration, an obvious sound energy gradient more easily forms between two adjacent lower- and higher-stage radiation surfaces, thus affecting the intensity and flow pathway of the acoustic streaming field. According to the acoustofluidic simulation results under different radiation surface combination modes, the calculated acoustic streaming patterns can be classified into three types. The first type comprises 1st + 4th RS, 1st + 2nd + 4th RS, 1st + 3rd + 4th RS, and 1st + 2nd + 3rd + 4th RS, and the acoustic streaming fields displayed by this kind of type are basically concentrated near the 1-stage radiation surfaces. The second type can be categorized as 2nd + 3rd RS, 2nd + 4th RS, 3rd + 4th RS, and 2nd + 3rd + 4th RS. In the case of the higher-stage radiation surfaces without the 1-stage radiation surfaces participating in vibration, the simulated acoustic streaming distributions are commonly strip-shaped and not concentrated around 1st RS. The third type can be classified as 1st + 2nd RS, 1st + 3rd RS, and 1st + 2nd + 3rd RS, and the computed acoustic streaming fields are both located near 1st RS and exhibit strip-shaped distributions. Generally speaking, with the introduction of multiple-stage radiation surfaces, the influence range of the local sound energy gradient expands, and the acoustic streaming velocity magnitude displayed by color bars also demonstrates that the more radiation surfaces are introduced, the larger the extreme value of fluid flow speed is. The particle trajectories successively plotted in Figure S4a (Circular cross-section) are generally consistent with the distribution characteristics of acoustic streaming patterns, which means that the Stokesian drag forces dominate the movement trajectories of polystyrene beads with the diameter of 1 μm [50].

However, the averaged acoustic streaming velocity magnitudes do not gradually increase with the sequential introduction of higher-stage radiation surfaces. It can be found from Figure 3c (Averaged acoustic streaming velocity magnitude) that when 4th RS, 2nd + 4th RS, 3rd + 4th RS, and 2nd + 3rd + 4th RS are combined with 1st RS, the corresponding fluid flow speeds are much smaller than before. The regularity is different from the increasing trend of the averaged acoustic streaming velocity magnitudes in the 3-stage quasi-Sierpiński-carpet-shaped chambers which have been published [39]. The reason for this phenomenon is that when the 1st RS participates in vibration, much more sound energy can be released than other higher-stage radiation surfaces. Therefore, a larger sound energy gradient can be preferentially formed among 1st RS and other vibration sources, resulting in the concentration of acoustic streaming vortices around 1st RS. Nevertheless, the sound energy generated by 1st RS will gradually dissipate when passing through the near-field 4-stage radiation surfaces, and the remaining ultrasonic waves transmitting to the far field and chamber periphery can compensate for the sound energy generated by other radiation surfaces, which narrows the range of sound energy gradient among multiple-stage radiation surfaces and weakens the far-field fluid flow speed, thus leading to the decrease of the overall averaged acoustic streaming velocity magnitude. The above-mentioned conclusion can also be confirmed by the simulation results shown in Figure 3b.

**Figure 4:**
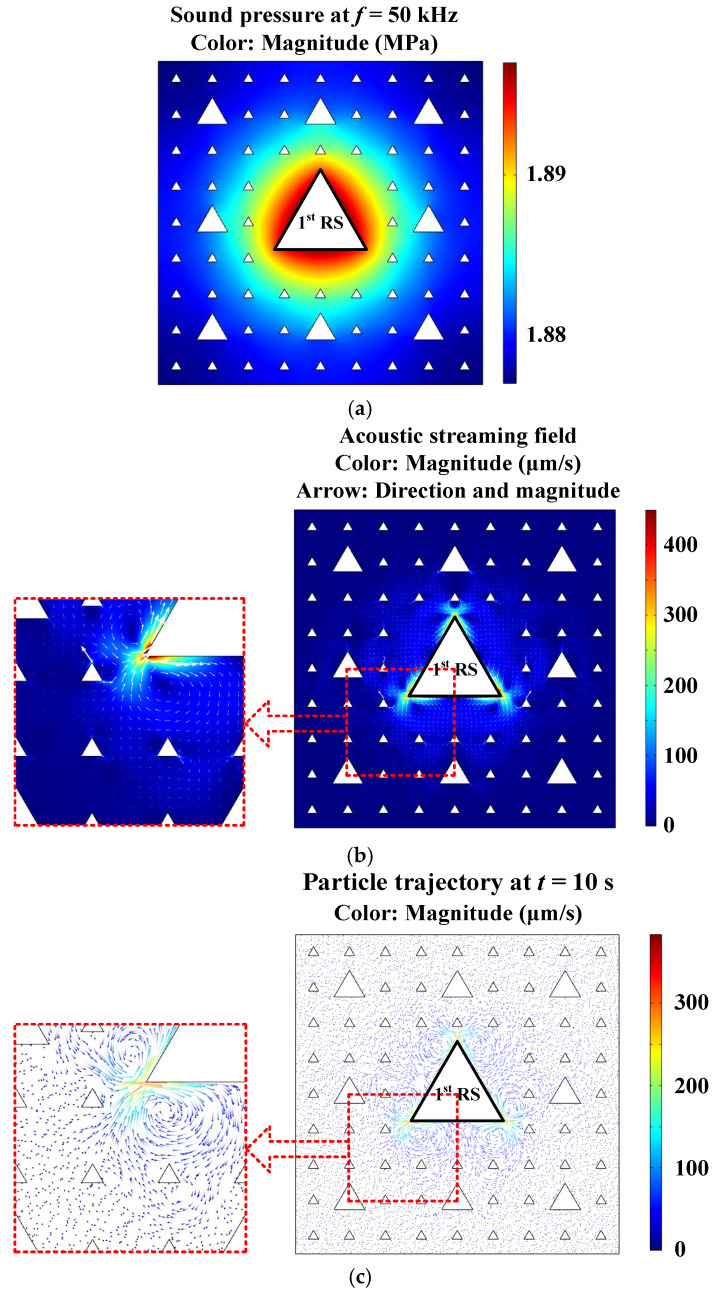
(Color online). Acoustofluidic fields and particle trajectories generated in the 3-stage pseudo-Sierpiński-carpet-shaped chamber with triangular cross-section under the excitation of 1st RS. (**a**) Pattern of sound pressure field. (**b**) Pattern of acoustic streaming field. (**c**) Pattern of micro particle trajectory at a given time (10 s).

Using the aforementioned construction method of the pseudo-fractal chamber, other kinds of chambers with diversified cross-sections of different geometric shapes have also been proposed, such as the 3-stage pseudo-Sierpiński-carpet-shaped chamber with equilateral triangular cross-section, as shown in Figure 4 (Acoustofluidic fields and particle trajectories generated in the 3-stage pseudo-Sierpiński-carpet-shaped chamber with triangular cross-section under the excitation of 1st RS). All the equilateral triangles are formed by the intersection of the corresponding circular cross-sections from Figure 1. Under the given boundary conditions and oscillation parameters, the sound field, acoustic streaming distribution and particle trajectory are calculated and respectively plotted from Figure 4a (Pattern of sound pressure field) to Figure 4c (Pattern of micro particle trajectory at a given time). The highlighted black lines still represent the 1-stage radiation surfaces (1st RS), and the other boundary conditions of acoustofluidic fields remain unchanged. The simulated sound field in Figure 4a is different from the one generated in the 3-stage pseudo-Sierpiński-carpet-shaped chamber with circular cross-section. Although it is still a bilateral symmetry pattern with discontinuous and uneven boundaries, a distribution mode similar to the Reuleaux triangle is presented.

Due to the existence of the sharp corners, local acoustic streaming vortices can be formed on both sides of each triangular corner, flowing in from the corner vertex and out from the edge midpoint, as shown in the red dashed box in Figure 4b (Pattern of acoustic streaming field), and the maximum acoustic streaming velocity magnitude in Figure 4b is approximately seven times that in Figure 1e. The comparative analysis and quantitative characterization of acoustofluidic fields generated in these two kinds of 3-stage pseudo- Sierpiński-carpet chambers with circular and triangular cross-sections reveals fundamentally distinct physical behaviors dictated by their geometric singularities. For the circular cross-section, the continuously differentiable contour facilitates a relatively uniform acoustic pressure distribution. The well-defined boundary layer along the smooth perimeter effectively dissipates energy, resulting in subdued acoustic streaming velocities near the circumferential region. In stark contrast, the triangular cross-section introduces geometric singularities at its vertices. The non-differentiable sharp corners disrupt the boundary layer continuity, leading to two synergistic effects: Enhanced Acoustic Energy Gradient, where a significantly steeper spatial gradient of acoustic energy is established between the vertices and edge midpoints of the primary radiation surface compared to the circular case due to the vibration magnification effect at the sharp corner; and Boundary Layer Ineffectiveness, where, at the vertices, the classical boundary layer formulation breaks down due to the geometric discontinuity, and therefore particle inertia dominates in these regions with minimal viscous damping effect from the boundary layer. On the other hand, at the sharp corners of the triangular cross-section, the first-order velocity variance increases sharply, resulting in the concentration of Reynolds stress, which significantly enhances local acoustic streaming. To sum up, the introduction of sharp corners will significantly strengthen the local acoustic streaming vortex effect. Also, the simulated particle movement trajectory plotted in Figure 4c can be used to verify the above conclusion.

**Figure 5:**
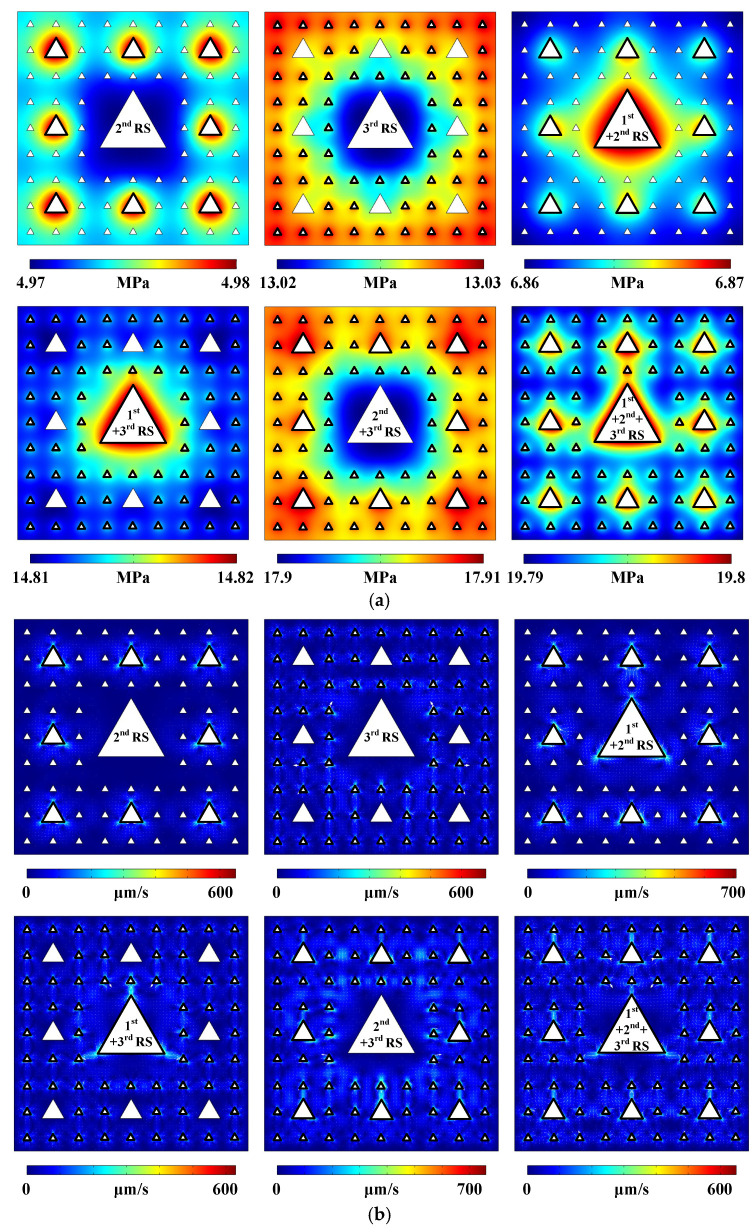

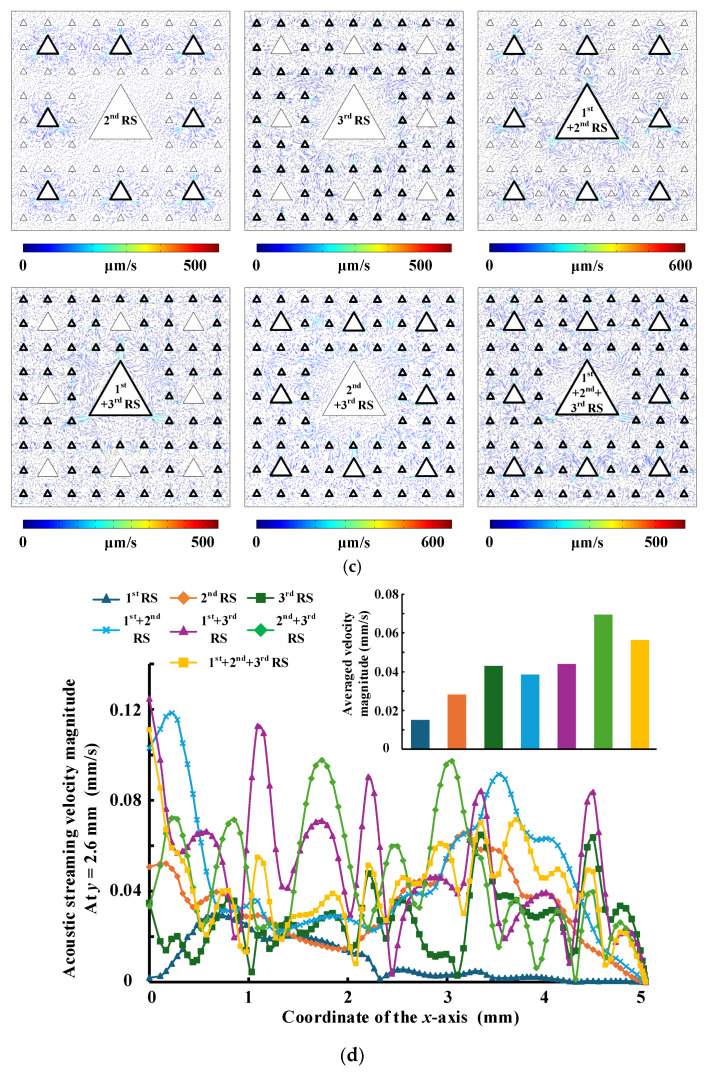
(Color online). Acoustofluidic fields and particle trajectories generated in the 3-stage pseudo-Sierpiński-carpet-shaped chamber with triangular cross-section under the excitation of different-stage radiation surfaces. (**a**) Pattern of sound pressure field. (**b**) Pattern of acoustic streaming field. (**c**) Pattern of micro particle trajectory at a given time (10 s). (**d**) Acoustic streaming velocity magnitude distribution.

The remaining sound fields under different radiation surface combination modes are also calculated and plotted in Figure 5a (Pattern of sound pressure field). When 1st RS and 2nd RS vibrate, the induced sound pressure patterns possess the fractalized characteristics of self-similarity and locality, while the extreme value region of the sound field generated by 3rd RS is mainly concentrated around the chamber peripheries. The sound field superposition modes under the combination of multiple radiation surfaces are almost consistent with the ones under the circumstances of the 3-stage pseudo/quasi-Sierpiński-carpet-shaped chambers with circular or square cross-section [39], except for the sound pressure distribution generated by 1st + 2nd + 3rd RS. When 1st + 2nd + 3rd RS are in operation, the extreme sound pressure region shown in the 3-stage pseudo-Sierpiński-carpet-shaped chamber with equilateral triangular cross-section is almost concentrated near 1st RS and 2nd RS, which differs from the results shown in Figure 2a. The reason for this phenomenon is that the extreme sound pressure region generated by 1st RS under the circumstance of equilateral triangular cross-section is concentrated around the three edge midpoints, and ultrasonic waves radiate outward from these three normal orientations. However, due to the long distances from the adjacent 2nd RS located in the corresponding transmission pathways or the cancellation effect of ultrasonic waves generated by the surrounding 3rd RS, obvious sound pressure patterns are mainly retained between 2nd RS and 3rd RS.

However, the acoustic streaming field distributions generated in the 3-stage pseudo-Sierpiński-carpet-shaped chamber with triangular cross-section still follow the fractal principle of self-similarity, as shown in Figure 5b (Pattern of acoustic streaming field). Regardless of the radiation surface combination mode, the concentrated regions of acoustic streaming mainly exist at the three sharp corners of each triangular cross-section, demonstrating the conclusion that the existence of sharp corners can amplify the vibration amplitude and significantly enhance the acoustic streaming effect, which is also consistent with our previously published work on quasi-Sierpiński-type fractal structures [39]. The sound energy gradients induced by different-stage radiation surfaces are almost concentrated around the individual sharp corners, rather than among different-stage solid–liquid interfaces, thus ensuring the self-similarity principle of acoustic streaming patterns. Particle trajectory images shown in Figure 5c (Pattern of micro particle trajectory at a given time) also indicate that polystyrene bead movement basically exists near the three sharp corners of each equilateral triangular cross-section.

Similarly, the acoustic streaming velocity magnitudes along the *x*-axis at *y* = 2.6 mm can be extracted from Figure 4b and Figure 5b and plotted as characteristic curves describing the acoustofluidic distributions, as shown in Figure 5d (Acoustic streaming velocity magnitude distribution). Due to the fact that the number of equilateral triangles corresponding to 3rd RS is the largest and the acoustic streaming vortices are mainly concentrated around the sharp corners, the extreme value numbers in the characteristic curves drawn with the participation of 3rd RS in vibration are usually larger than those drawn with only 1st RS or 2nd RS. Assuming that the height of the pseudo-Sierpiński-carpet-shaped chamber is *H*, the total areas of multiple-stage radiation surfaces in Figure 5 (Acoustofluidic fields and particle trajectories generated in the 3-stage pseudo-Sierpiński-carpet-shaped chamber with triangular cross-section under the excitation of different-stage radiation surfaces) can be expressed as $\sum_{n}\frac{\sqrt{3}}{10}\left[{\left(\frac{8}{3}\right)}^{n}-1\right]L_{0}H$. Therefore, the area ratio of 1st RS, 2nd RS, 3rd RS, 1st + 2nd RS, 1st + 3rd RS, 2nd + 3rd RS, and 1st + 2nd + 3rd RS is 9:24:64:33:73:88:97. However, the averaged acoustic streaming velocity magnitudes corresponding to the inserted image in Figure 5d is about 15:27:42:38:44:68:55, which does not match the above area ratio, indicating that there exist inhibitory effects among the chaotic and complex acoustic streaming vortices generated by excessive sharp corner structures and overly dense radiation surfaces.

**Figure 6:**
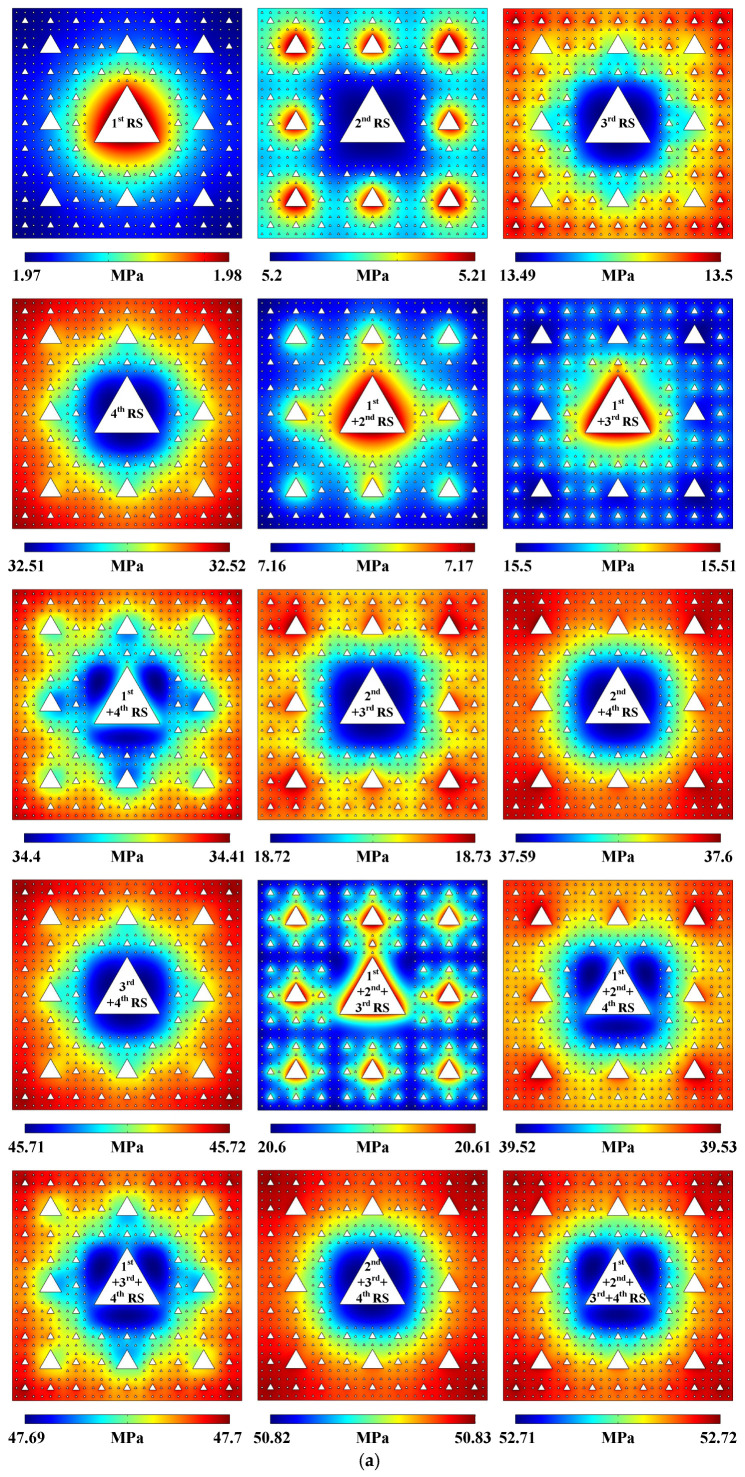

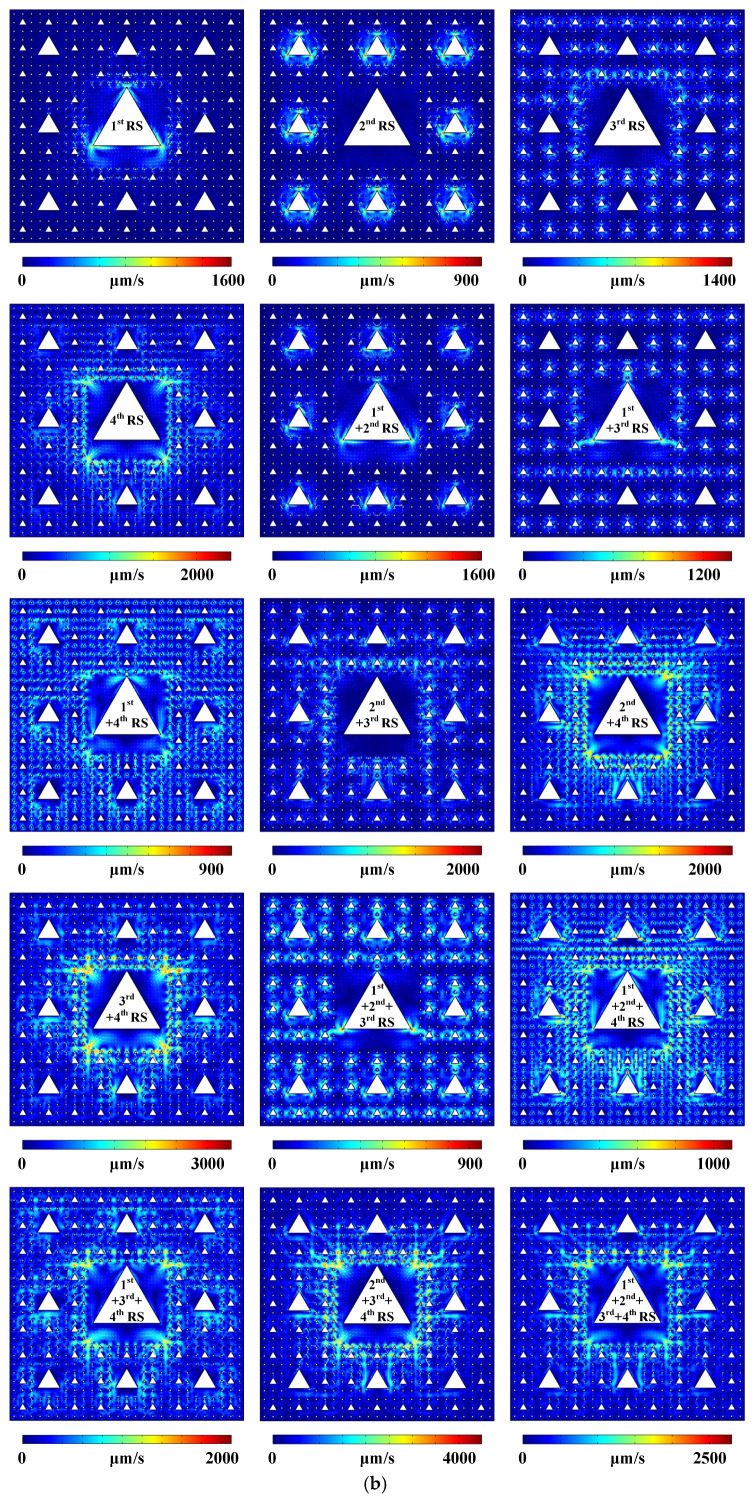

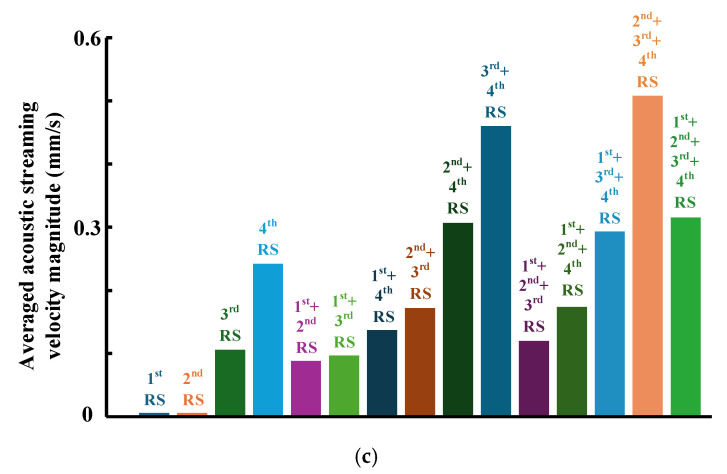
(Color online). Acoustofluidic fields and particle trajectories generated in the 4-stage pseudo-Sierpiński-carpet-shaped chamber with triangular cross-section under the excitation of different-stage radiation surfaces. (**a**) Pattern of sound pressure field. (**b**) Pattern of acoustic streaming field. (**c**) Averaged acoustic streaming velocity magnitude.

According to simulation results shown in Figure 6a (Pattern of sound pressure field), the sound pressure extreme values activated by 1st RS or 2nd RS are mainly concentrated in the vicinity of 1st RS or 2nd RS, respectively, while the extreme regions induced by the vibration of 3rd RS or 4th RS are concentrated around the peripheries of the 4-stage pseudo-Sierpiński-carpet-shaped chamber, among which the one generated by 4th RS is more widely distributed. Unlike the sound pressure patterns in the 4-stage pseudo-Sierpiński-carpet-shaped chamber with circular cross-section, the existence of 4th RS under the circumstance of triangular cross-section possesses the strongest influence on the sound field distribution, followed by 1st RS, then 3rd RS, and finally 2nd RS. Also, the sound field patterns under different combination modes of multiple-stage vibration sources are mainly determined by the individual radiation surfaces with the corresponding strongest influence mentioned above. For instance, the sound field distributions generated by the combination modes with 4th RS participating in oscillation are basically similar to the simulation result of individual 4th RS, while the sound pressure patterns activated by the combination modes without 4th RS but with 1st RS participating in oscillation are predominantly concentrated in the vicinity of 1st RS.

The introduction of 4th RS leads to slight differences in the acoustic streaming distributions generated in the 4-stage pseudo-Sierpiński-carpet-shaped chambers with equilateral triangular cross-section, which is due to the fact that 4th RS are densely arranged near other vibration sources. The acoustic waves radiated outward from the sharp corners of the lower-stage radiation surfaces and the ones reflected back from the boundaries of the surrounding 4th RS can superimpose to form a strong standing wave field, enhancing the sound energy gradients at the sharp corners of the lower-stage radiation surfaces and the resulting acoustofluidic effect, as shown by the acoustic streaming vortex patterns generated under the circumstances of individual or combined 1st RS, 2nd RS, and 3rd RS in Figure 6b (Pattern of acoustic streaming field). However, the acoustic streaming fields induced by the introduction of 4th RS present radial-like distributions along the four corners of the square chamber, which is due to the fact that there exist massive 4-stage radiation surfaces around each chamber corner, and the generated acoustic waves propagate, reflect and diffract among these solid–liquid interfaces, thus forming intense standing wave fields inside the entire chamber. Meanwhile, since the sound energy gradients near the chamber corners are relatively larger, radial-like acoustic streaming patterns can be formed. Similar to the above-mentioned conclusion drawn from the sound pressure distributions, all the combination modes with 4th RS exhibit acoustic streaming patterns similar to that generated by individual 4th RS, followed by 1st RS, then 3rd RS, and finally 2nd RS. For example, if the acoustic streaming fields are activated by the combination modes without 4th RS but with 1st RS, the vortex patterns are similar to those generated by individual 1st RS. The particle movement trajectories plotted in Figure S4b (Triangular cross-section) can also be used to confirm the above distributions of acoustic streaming fields.

Figure 6c (Averaged acoustic streaming velocity magnitude) shows the variation regularity of the averaged acoustic streaming velocity magnitudes under different radiation surface combination modes in the 4-stage pseudo-Sierpiński-carpet-shaped chamber with equilateral triangular cross-section, which is similar to the one presented under the situation of circular cross-section. Once 1st RS are introduced into combination modes with 3rd RS or 4th RS, the averaged acoustic streaming velocity magnitudes will decrease, and the decrease degrees will be even larger in the presence of 4th RS, which is due to the fact that the arrangement of 4th RS is much denser than that of 3rd RS, releasing more sound energy and stimulating wider acoustic streaming range, thus offsetting the acoustic streaming velocity magnitudes excited by 1st RS to a greater extent.

**Figure 7:**
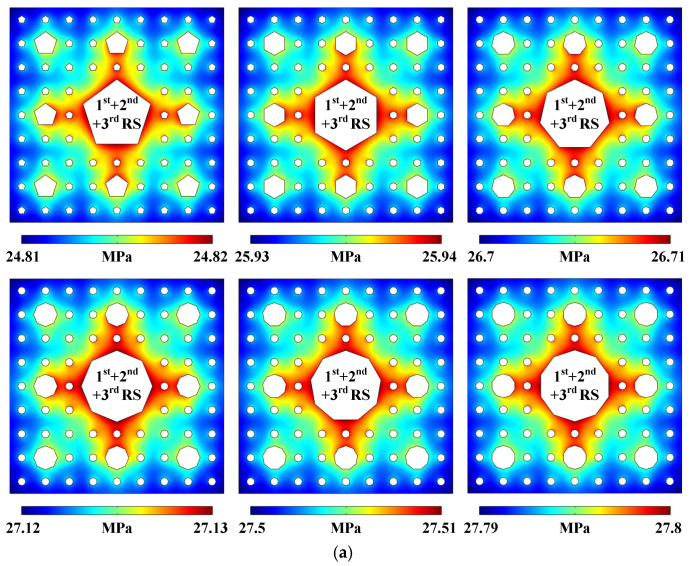

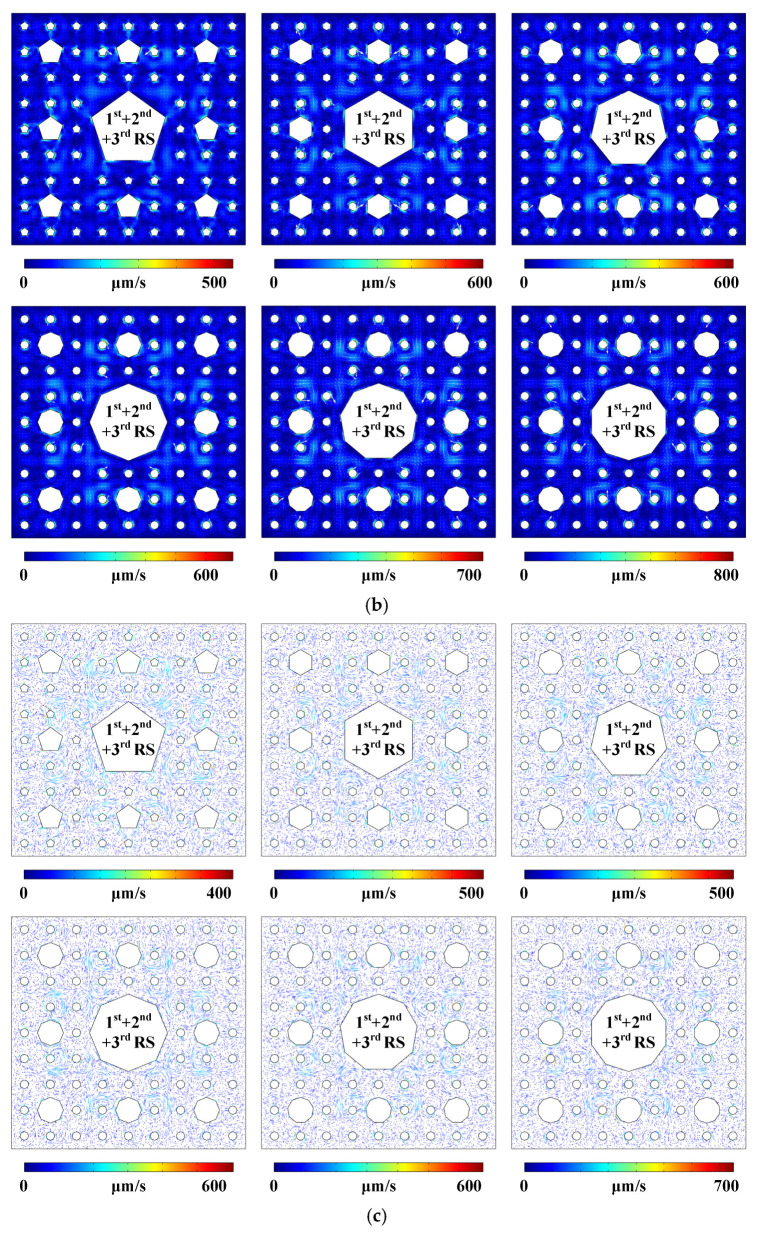

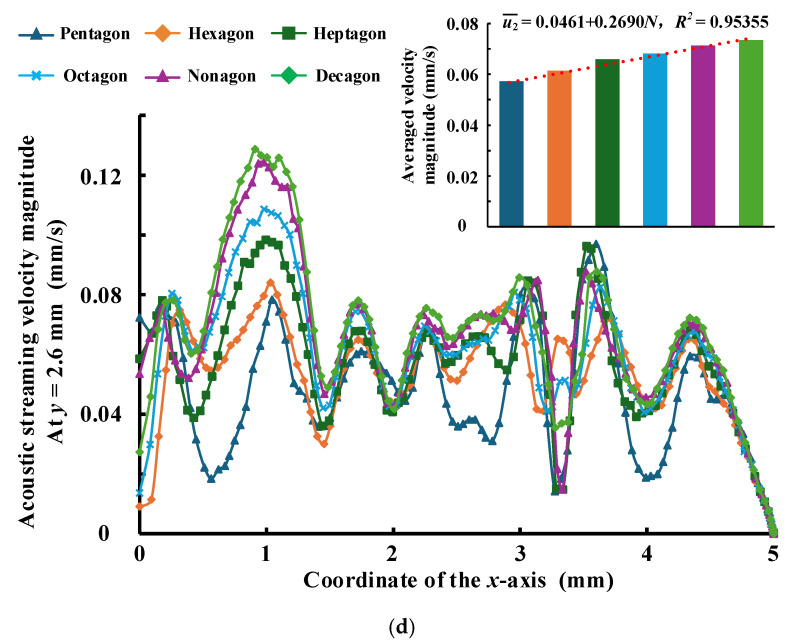
(Color online). Acoustofluidic fields and particle trajectories generated in the 3-stage pseudo-Sierpiński-carpet-shaped chamber with different regular polygonal cross-sections under the excitation of 1st + 2nd + 3rd RS. (**a**) Pattern of sound pressure field. (**b**) Pattern of acoustic streaming field. (**c**) Pattern of micro particle trajectory at a given time (10 s). (**d**) Acoustic streaming velocity magnitude distribution.

In order to explore more distributions of sound fields, acoustic streaming vortices, and particle motion trajectories under the circumstances of different regular polygonal cross-sections, the following simulation results are simplified by applying the same radiation surface combination mode. Similar to the sound pressure distribution generated by 1st + 2nd + 3rd RS in the 3-stage pseudo-Sierpiński-carpet-shaped chamber with circular cross-section, the ones shown in Figure 7a (Pattern of sound pressure field) induced by the same radiation surface combination mode corresponding to different cross-sectional configurations from regular pentagon to regular decagon inscribed within the corresponding circular cross-section apparently exhibit concave square patterns rotated by 45°, and the main difference, excluding the magnitude factor, is the concavity degree of the square distributions, which gradually decreases with the increase of regular polygonal edge number (defined as *N*) and also tends to approach the sound field generated by 1st + 2nd + 3rd RS under the situation of circular cross-section. According to the construction method of regular polygons, it can be found that as the edge number *N* increases, the radiation surface shapes and areas of regular polygonal cross-sections tend to tend to approach those under the circumstance of a circular cross-section. Therefore, the generated sound pressure pattern plotted in Figure 7a becomes gradually similar to the last one presented in Figure 2a.

The acoustic streaming distributions also present the same changing principle as the sound fields. With the increase of *N*, the generated acoustofluidic patterns are progressively approaching the pattern generated by 1st + 2nd + 3rd RS under the situation of circular cross-section. However, considering the circular cross-section is continuous and smooth, the flow resistance of the liquid–solid interfaces to the acoustic streaming field is the smallest in comparison with other cross-sectional configurations. For regular polygons with fewer *N*, the sharp corner degrees are relatively smaller, and the acoustofluidic vortices are almost concentrated around each corner, while for regular polygons with more edges, the corner effect is no longer obvious. Therefore, as the regular polygonal edge number increases, the strip-shaped acoustofluidic distributions are more distinctly displayed in Figure 7b (Pattern of acoustic streaming field). The particle movement trajectories under different cross-sectional configurations plotted in Figure 7c (Pattern of micro particle trajectory at a given time) also demonstrate the same regularity as described above.

According to the characteristic curves at *y* = 2.6 mm extracted from Figure 7b and shown in Figure 7d (Acoustic streaming velocity magnitude distribution), it can be found that except for the different extreme value magnitudes, the variation trends and extreme value numbers of each characteristic curve are basically consistent. Especially from *x* = 4.5 mm to *x* = 5 mm, the coincidence degree of the six characteristic curves is relatively high, which is due to the fact that this region is far from 1st RS and predominantly affected by 3rd RS. The above results also indicate that the number and position of acoustic streaming vortices along the extracted characteristic curve pathways under the circumstances of six cross-sectional configurations are almost the same. Moreover, with the increase of *N*, the extreme value magnitude gradually approaches the one generated by the situation of circular cross-section. The averaged acoustic streaming velocity magnitude shown in the inserted image (see Figure 7d) is also related to the regular polygonal edge number. As *N* increases, the radiation surface area and the released sound energy also increase, resulting in an increase in the averaged fluid flow speed throughout the entire chamber. The red dotted line plotted in the inserted image represents a fitting curve based on the six magnitude values, which can be written as $\bar{u_{2}}=0.0461+0.2690N$, and the coefficient of determination *R*^2^ is about 0.95355, indicating a high degree of linear correlation.

**Figure 8:**
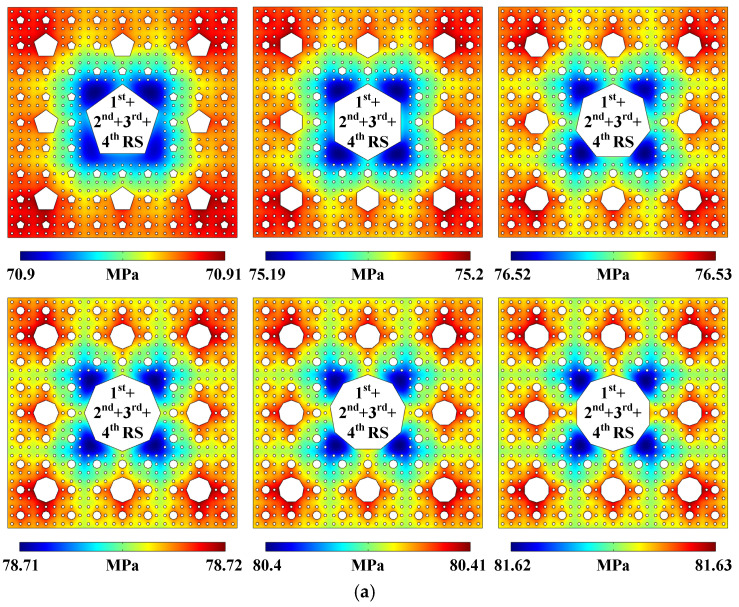

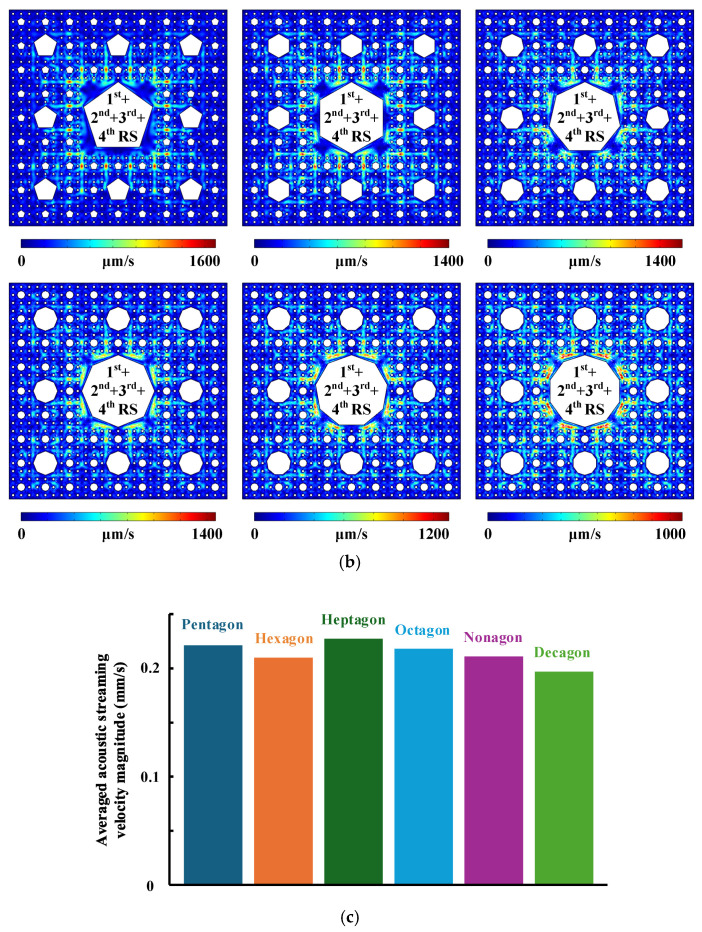
(Color online). Acoustofluidic fields and particle trajectories generated in the 4-stage pseudo-Sierpiński-carpet-shaped chamber with different regular polygonal cross-sections under the excitation of 1st + 2nd + 3rd + 4th RS. (**a**) Pattern of sound pressure field. (**b**) Pattern of acoustic streaming field. (**c**) Averaged acoustic streaming velocity magnitude.

More sound pressure distributions generated by 1st + 2nd + 3rd + 4th RS in the 4-stage pseudo-Sierpiński-carpet-shaped chambers with different regular polygonal cross-sections are plotted in Figure 8a (Pattern of sound pressure field). As *N* increases, the extreme sound pressure regions progressively converge from the chamber periphery to the vicinity of the 2nd RS, and the cross-shaped sound pressure patterns gradually form between 1st RS and 2nd RS, approaching the situation of circular cross-section. The acoustic streaming fields presented in Figure 8b (Pattern of acoustic streaming field) are all strip-shaped, while the acoustofluidic vortices which are originally concentrated around the sharp corners of the 1-stage regular polygonal cross-section will gradually shift to the vicinity of 1st RS and spread towards the chamber periphery with the increase of *N*, positively relating to the increase in the degree of sharp corners and the total area of radiation surfaces. The particle trajectories shown in Figure S5 (Particle trajectories generated in the 4-stage pseudo-Sierpiński-carpet-shaped chamber with different regular polygonal cross-sections under the excitation of 1st + 2nd + 3rd + 4th RS) also validate the above regularity. The averaged acoustic streaming velocity magnitudes in the 4-stage pseudo-Sierpiński-carpet-shaped chambers with different regular polygonal cross-sections can be calculated and shown in Figure 8c (Averaged acoustic streaming velocity magnitude), and the variation tendency of fluid flow speed is also related to the sharp corner degree and the radiation surface area. The angle increase will weaken the acoustofluidic amplification effect at each sharp corner, while the area increase will enhance the acoustic streaming field. Therefore, the averaged acoustic streaming velocity magnitude reaches the maximum value under the circumstance of regular heptagonal cross-section.

More simulation results of the acoustofluidic fields and particle movement trajectories generated in the 3- and 4-stage pseudo-Sierpiński-carpet-shaped chambers with other cross-sections (i.e., Reuleaux polygon, pentagram, and concave square) are listed in the Supplementary Material as shown from Figure S6 (Acoustofluidic fields and particle trajectories generated in the 3-stage pseudo-Sierpiński-carpet-shaped chamber with different Reuleaux polygonal cross-sections under the excitation of 1st + 2nd + 3rd RS) to Figure S13 (Acoustofluidic fields and particle trajectories generated in the 4-stage pseudo-Sierpiński-carpet-shaped chamber with concave square cross-section under the excitation of different-stage radiation surfaces). Also, the averaged acoustic streaming velocity magnitudes under all circumstances are consolidated and presented in Table S1 (Averaged acoustic streaming velocity magnitude under all circumstances). Since the generation mechanism and evolution principle of diversified acoustofluidic distributions have been illuminated in the above-mentioned main sections, no more detailed descriptions are included in the Supplementary Material.

In summary, although the combination of multiple radiation surfaces can linearly superimpose sound pressure amplitudes, the distribution of the acoustic energy gradient is modified through a nonlinear mechanism, ultimately affecting the morphology and intensity of the acoustic streaming field. Therefore, the linear acoustic field establishes the fundamental framework for energy distribution, while the nonlinear acoustic streaming effect gives rise to rich steady-state flow modes within this framework, collectively enabling controllable modulation of particle motion trajectories. The coupling relationship between the two mechanisms can be summarized as follows: the linear acoustic field determines the distribution of energy emission through boundary conditions, while the nonlinear acoustofluidic effect converts sound energy gradient into fluid kinetic momentum through an inherent nonlinear mechanism. Therefore, the heterogeneous acoustic streaming distributions and particle movement trajectories achieved in this study are essentially a vivid manifestation of the nonlinear acoustofluidic effect output indirectly programmed by designing the spatial energy distribution of the linear sound field (i.e., geometric and radiation surface arrangement). This clear distinction and correlation provide profound physical guidance for the design of acoustofluidic devices based on fractal or complex geometries; that is, by manipulating the boundary conditions of linear sound fields, the functional output of nonlinear acoustic streaming vortices can be predictively shaped.

In comparison to existing studies on fractal acoustics or acoustofluidics, this work introduces pseudo-fractal structures (such as pseudo-Sierpiński-carpet) characterized by regularity yet lacking strict self-similarity in geometric design. Such structures leverage the spatial symmetry and multi-scale nesting features of the given geometry to effectively modulate the distribution of both acoustic pressure distributions and acoustic streaming fields without relying on complex parametric adjustments. The study further reveals that although the pattern of acoustic streaming fields does not fully adhere to the self-similarity of fractals, their formation still obeys the principles of energy conservation and linear superposition. This suggests that in the design of fractal-inspired acoustofluidic devices, the geometric arrangement and excitation combinations of radiation surfaces can be flexibly tailored to customize acoustic streaming vortex patterns. Additionally, the cross-sectional shape exhibits a significant influence on the enhancement of acoustic streaming effects, particularly for geometries with sharp edges (such as triangular cross-section), where boundary layer discontinuity and concentrated acoustic energy gradients markedly amplify acoustic streaming magnitude. However, its limitation lies in the fact that due to the absence of strict adherence to the fractal self-similarity principle, the acoustic pressure distribution does not fully exhibit fractal characteristics. Moreover, the dense arrangement of higher-order radiation surfaces may lead to mutual suppression among acoustic streaming vortices, thereby affecting the overall acoustic streaming intensity. Distinct from conventional construction methods of miniaturized microfluidic systems, the artificial introduction of fractal elements such as Sierpiński carpets/triangles, Koch snowflakes, Mandelbrot sets, and Pythagorean trees offers novel perspectives and expands the application scope of acoustofluidic effects, simultaneously enriching and diversifying ultrasonic micro/nano-scale manipulation technologies. By incorporating fractal-inspired multi-scale geometric structures and independently controllable radiation surface arrays, this approach provides a new methodology for the spatial customization of acoustic field energy and acoustic streaming distribution.

## 4. Conclusions

In this work, a novel strategy for generating heterogeneous distributions of patterned acoustofluidic fields in a series of pseudo-Sierpiński-carpet-shaped chambers with various cross-sectional geometries and ultrasonic excitation conditions is proposed and computed. All of the radiation surfaces located in the pseudo-fractal chambers possess the same settings of input frequency point, oscillation amplitude, and initial phase distribution along individual normal orientation. The simulation results of sound pressure fields, acoustic streaming patterns and particle motion trajectories show that the diversified geometric radiation surfaces in the pseudo-Sierpiński-carpet-shaped chambers can feasibly realize customized manipulation or rapid amalgamation of abundant particles/bio-samples. In comparison with our published paper, the acoustofluidic distributions and particle movement trajectories actuated by the pseudo-fractal configurations do not strictly follow the characteristic of self-similarity, while the generation of acoustic streaming vortices still follow the internal principles of energy conservation and linear superposition, which broadens the application prospect of ultrasonic micro/nano-scale manipulation technology. It is undeniable that although the current work is primarily a proof-of-concept numerical study, its core value lies in validating the feasibility and potential of introducing fractal element technology or method in practical acoustofluidic applications through theoretical modeling and simulation, which provides a basis for the implementation of subsequent experimental verification and engineering deployment. The possible fabrication methodology for the 3D pseudo-Sierpiński-carpet-shaped acoustic chamber and the schematic diagram of the anticipated vibration mechanism are provided in the Supplementary Material. The chamber structure can be manufactured via established techniques such as soft lithography or micro-scale 3D printing. Piezoelectric films or layers are integrated onto the top surface of each hierarchical stage of the chamber. The vertical oscillation induced by each piezoelectric element can be transduced into normal vibration of the corresponding radiation surface, primarily mediated by the Poisson effect within the chamber stage material. Also, in subsequent studies, integrating more complex fractal geometries with phononic crystal structures could allow the customization of acoustofluidic fields with higher spatial complexity and functionality, thereby providing a novel technical platform for morphological manipulation of biological samples, investigation of single-cell mechanical properties, and directional actuation and assembly of micro/nano-scale machines.

## Figures and Tables

**Table 1:** Model parameters in the simulation.

Parameter	Abbreviation	Value	Unit
Initial side length of chamber	*L*_0_	10	mm
Normal vibration amplitude	*A*	100	nm
Input vibration frequency point	*f*	50	kHz
Initial phase distribution	*θ*_0_	0	rad
Maximum element size of all grids	*L_G_*	0.1	mm
Density of water	*ρ*_0_	1000	kg/m^3^
Sound speed in water	*c*_0_	1500	m/s
Shear viscosity of water	*μ*	0.001	Pa·s
Volume-to-shear viscosity ratio of water	*μ_b_*/*μ*	2.79	1
Heat capacity at constant pressure of water	*C_P_*	4200	J/(kg·K)
Heat conductivity coefficient of water	*k*	0.6	W/(m·K)
Density of polystyrene beads	*ρ_p_*	1050	kg/m^3^
Pressure-wave speed in polystyrene beads	*c_p_*	2400	m/s
Shear-wave speed in polystyrene beads	*c_s_*	1150	m/s
Diameter of polystyrene beads	*d_p_*	1	μm

## Data Availability

The raw data supporting the conclusions of this article will be made available by the corresponding author on request.

## Supplementary Materials

The following supporting information can be downloaded at: https://www.mdpi.com/article/10.3390/mi17020259/s1, Figure S1: Schematic diagram of fabrication method and oscillation mode; Figure S2: Driving force magnitudes generated in the 3-stage pseudo-Sierpiński-carpet-shaped chamber with circular cross-section under the excitation of 1st RS; Figure S3: Acoustofluidic fields generated in the 3-stage pseudo-Sierpiński-carpet-shaped chamber with circular cross-section under the excitation of different-stage radiation surfaces with different initial phase settings; Figure S4: Particle trajectories generated in the 4-stage pseudo-Sierpiński-carpet-shaped chamber with different cross-sections under the excitation of different-stage radiation surfaces; Figure S5: Particle trajectories generated in the 4-stage pseudo-Sierpiński-carpet-shaped chamber with different regular polygonal cross-sections under the excitation of 1st + 2nd + 3rd + 4th RS; Figure S6: Acoustofluidic fields and particle trajectories generated in the 3-stage pseudo-Sierpiński-carpet-shaped chamber with different Reuleaux polygonal cross-sections under the excitation of 1st + 2nd + 3rd RS; Figure S7: Acoustofluidic fields and particle trajectories generated in the 4-stage pseudo-Sierpiński-carpet-shaped chamber with different Reuleaux polygonal cross-sections under the excitation of 1st + 2nd + 3rd + 4th RS; Figure S8: Acoustofluidic fields and particle trajectories generated in the 3-stage pseudo-Sierpiński-carpet-shaped chamber with pentagrammal cross-section under the excitation of 1st RS; Figure S9: Acoustofluidic fields and particle trajectories generated in the 3-stage pseudo-Sierpiński-carpet-shaped chamber with pentagrammal cross-section under the excitation of different-stage radiation surfaces; Figure S10: Acoustofluidic fields and particle trajectories generated in the 4-stage pseudo-Sierpiński-carpet-shaped chamber with pentagrammal cross-section under the excitation of different-stage radiation surfaces; Figure S11: Acoustofluidic fields and particle trajectories generated in the 3-stage pseudo-Sierpiński-carpet-shaped chamber with concave square cross-section under the excitation of 1st RS; Figure S12: Acoustofluidic fields and particle trajectories generated in the 3-stage pseudo-Sierpiński-carpet-shaped chamber with concave square cross-section under the excitation of different-stage radiation surfaces; Figure S13: Acoustofluidic fields and particle trajectories generated in the 4-stage pseudo-Sierpiński-carpet-shaped chamber with concave square cross-section under the excitation of different-stage radiation surfaces; Table S1: Averaged acoustic streaming velocity magnitude (μm/s) under all circumstances.
