# Supplementary material for: Heterogeneous Acoustofluidic Distributions Induced by Different Radiation Surface Arrangements in Various Pseudo-Sierpiński-Carpet-Shaped Chambers
Source: Micromachines (Basel). 2026 Feb 16;17(2):259. doi: 10.3390/mi17020259 (PMC12943372; doi:10.3390/mi17020259)
Supplement: Supplementary file 1 [file micromachines-17-00259-s001.zip › micromachines-4124949-supplementary.pdf]

micromachines

Supplementary material

Heterogeneous acoustofluidic distributions induced by different radiation surface arrangements in various pseudo-Sierpiński-carpet shaped chambers

Qiang Tang [1,*], Boyang Li [1], Chen Li [1], Junjie Wang [1], Huiyu Huang [1], Yulong Hu [1], Kan Zhu [1], Hao Chen [1], Xu Wang [2] and Songfei Su [3]

[1] Jiangsu Key Laboratory of Advanced Manufacturing Technology, Faculty of Mechanical and Material Engineering, Huaiyin Institute of Technology, Huaian 223003, China; m18205233053@163.com (B.L.); lc924938166@163.com (C.L.); curry_2026@foxmail.com (J.W.); hyhuang@hyit.edu.cn (H.H.); 15150706701@163.com (Y.H.); zhukan860109@hyit.edu.cn (K.Z.); chenhao@hyit.edu.cn (H.C.)
[2] College of Mechanical and Electronic Engineering, Nanjing Forestry University, Nanjing 210037, China; schuewang@njfu.edu.cn
[3] School of Mechanical Engineering, Nanjing Institute of Technology, Nanjing 211167, China; susongfeinh@163.com
* Correspondence: tangqiang102@126.com; Tel.: +86 15151845559

Academic Editor: Firstname Lastname

Received: date
Revised: date
Accepted: date
Published: date

Citation: To be added by editorial staff during production.

Copyright: © 2025 by the authors. Submitted for possible open access publication under the terms and conditions of the Creative Commons Attribution (CC BY) license (https://creativecommons.org/licenses/by/4.0/).

1. Fabrication method and oscillation mode of 3D pseudo-Sierpiński-carpet shaped chamber

Figure S1 illustrates the fabrication method of 3D pseudo-Sierpiński-carpet-shaped acoustic chamber and schematically depicts the possible vibration mechanism. The chamber can be fabricated using conventional soft lithography or 3D micro-printing techniques. Piezoelectric films/layers can be integrated onto the upper surface of each chamber stage by referring the existing literature [1]. The vertical oscillation generated by each piezoelectric film/layer are subsequently converted into normal vibration of the corresponding radiation surface through the Poisson effect.

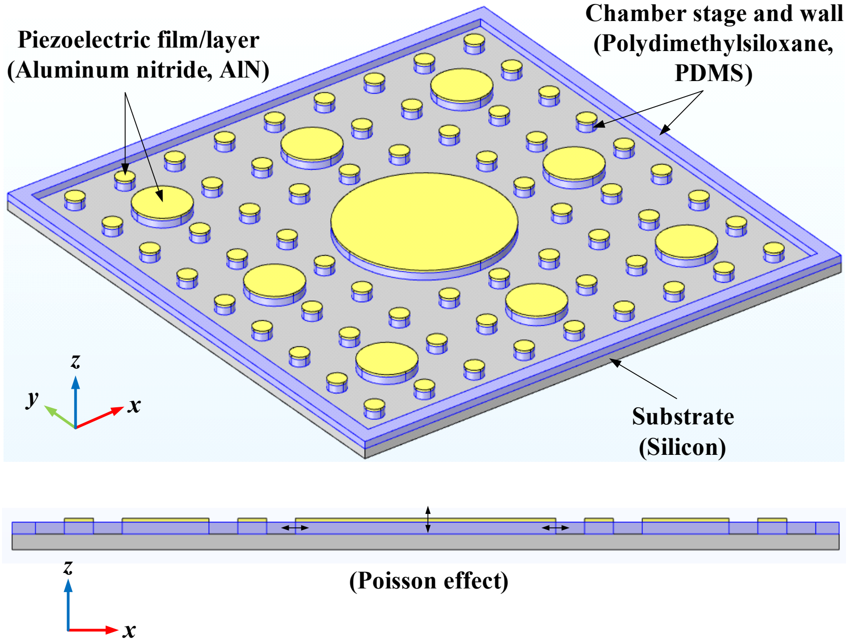

Figure S1. (Color online). Schematic diagram of fabrication method and oscillation mode.

2. Comparison among the magnitudes of acoustic radiation force, acoustic streaming induced drag force, gravity force, and buoyancy force

From Eqs. 12 and 13, the magnitude of acoustic radiation force can be calculated by $\|\boldsymbol{F}_{rad}\| = \sqrt{(F^{x}_{rad})^2 + (F^{y}_{rad})^2} = \frac{4}{3}\pi R_p^3 \left\| \nabla[\frac{1-\beta}{2\rho_0 c_0^2}\overline{p_1^2} - \frac{D}{2}\rho_0 \overline{\|\boldsymbol{u}_1\|^2}] \right\|$, and the magnitude of initial acoustic streaming induced drag force magnitude can be calculated by $\|\boldsymbol{F}_{drag}\| = \sqrt{(F^{x}_{drag})^2 + (F^{x}_{drag})^2} = 6\pi\mu R_p \|\boldsymbol{u}_2\|$ [2]. By using the post-processing function of COMSOL Multiphysics, the patterns of the acoustic radiation force magnitude and the initial acoustic streaming induced drag force magnitude acting on polystyrene beads with a diameter of 1 μm generated in the 3-stage pseudo-Sierpiński-carpet shaped chamber with circular cross-section under the excitation of 1st RS can be plotted in Fig. S2. Also, according to the definition, the calculated magnitude of gravity force ($\boldsymbol{F}_{gravity} = \rho_p \frac{4}{3}\pi R_p^3 g$) and buoyancy force ($\boldsymbol{F}_{buoyancy} = \rho_0 \frac{4}{3}\pi R_p^3 g$) acting on the particles is about 4.3×10^{-14} N and 4.1×10^{-14} N, respectively, which is smaller than that of the driving force in the acoustofluidic field, and can therefore be neglected in the simulation.

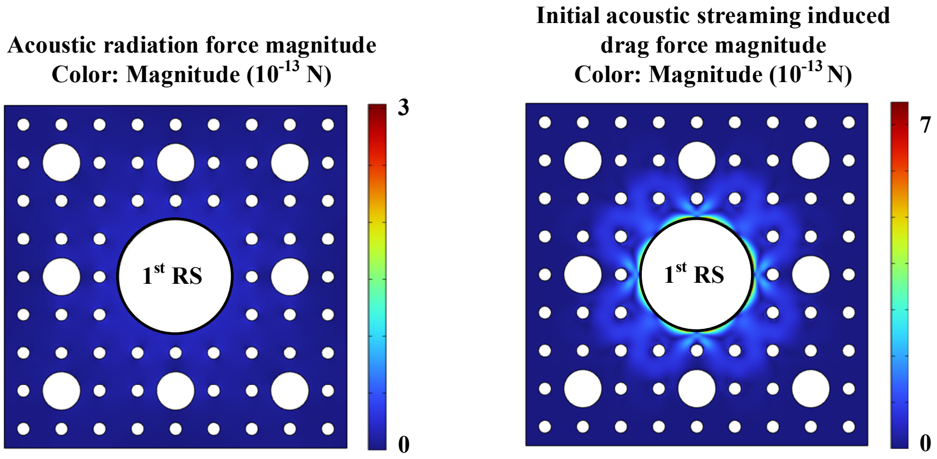

(a) (b)

Figure S2. (Color online). Driving force magnitudes generated in the 3-stage pseudo-Sierpiński-carpet shaped chamber with circular cross-section under the excitation of 1st RS. **(a)** Acoustic radiation force magnitude. **(b)** Initial acoustic streaming induced drag force magnitude.

3. Preliminary calculation and influence comparison of different initial phase settings on acoustofluidic fields

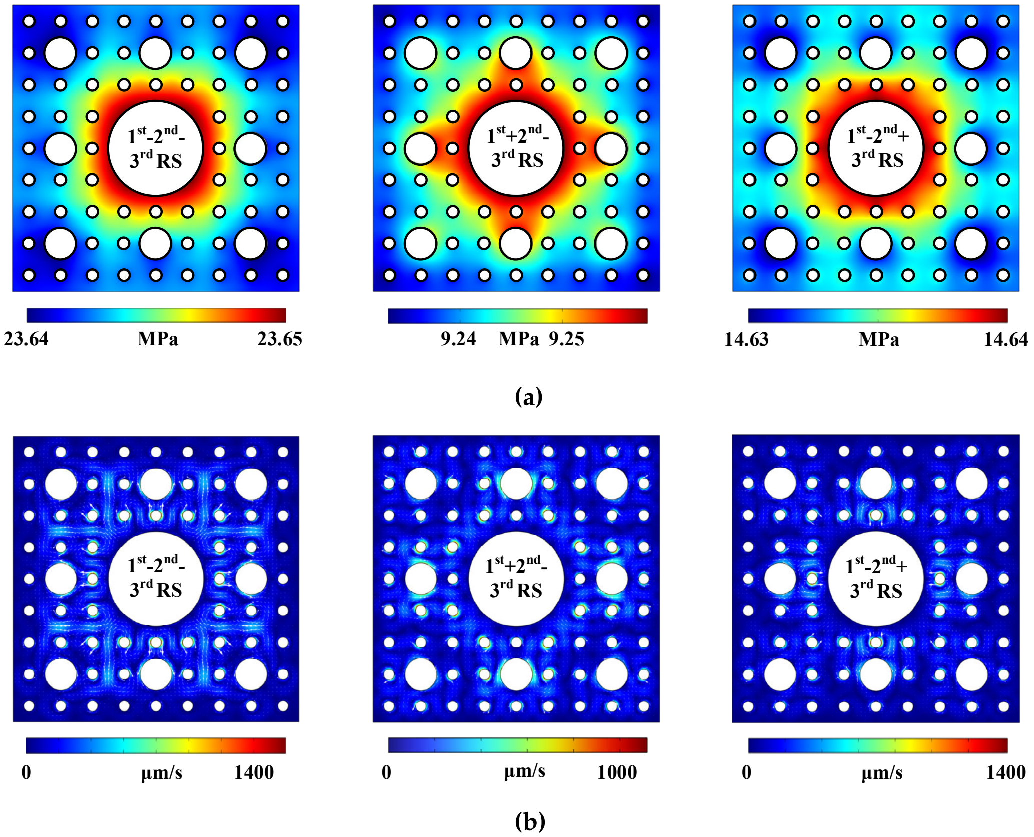

Figure S3. (Color online). Acoustofluidic fields generated in the 3-stage pseudo-Sierpiński-carpet shaped chamber with circular cross-section under the excitation of different-stage radiation surfaces with different initial phase settings. **(a)** Pattern of sound pressure field. **(b)** Pattern of acoustic streaming field.

4. Particle trajectories generated in the 4-stage pesudo-Sierpiński-carpet shaped chambers with different cross-sections under the excitation of different-stage radiation surfaces

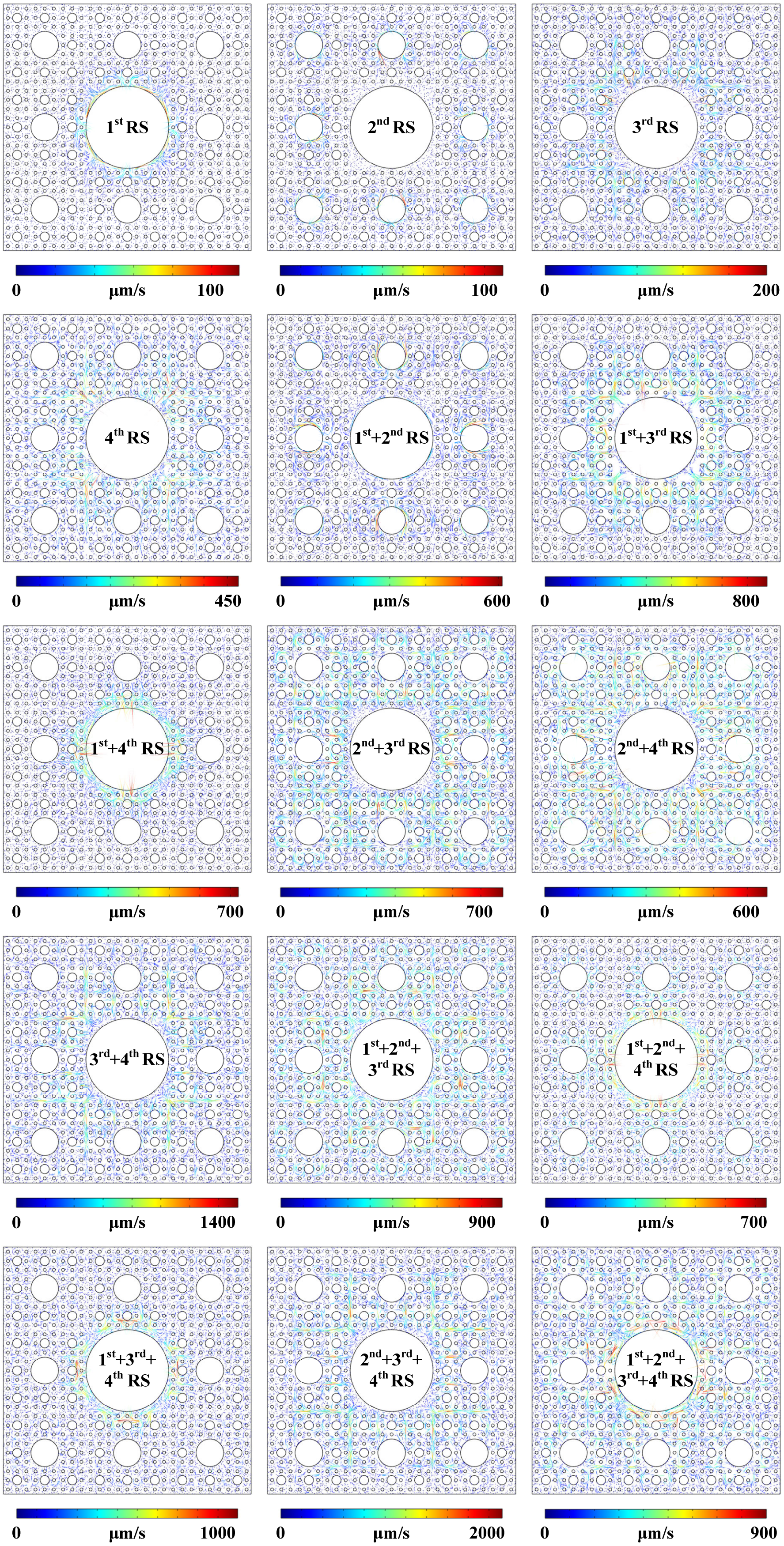
1st RS
0 μm/s 100
2nd RS
0 μm/s 100
3rd RS
0 μm/s 200
4th RS
0 μm/s 450
1st+2nd RS
0 μm/s 600
1st+3rd RS
0 μm/s 800
1st+4th RS
0 μm/s 700
2nd+3rd RS
0 μm/s 700
2nd+4th RS
0 μm/s 600
3rd+4th RS
0 μm/s 1400
1st+2nd+3rd RS
0 μm/s 900
1st+2nd+4th RS
0 μm/s 700
1st+3rd+4th RS
0 μm/s 1000
2nd+3rd+4th RS
0 μm/s 2000
1st+2nd+3rd+4th RS
0 μm/s 900

(a)

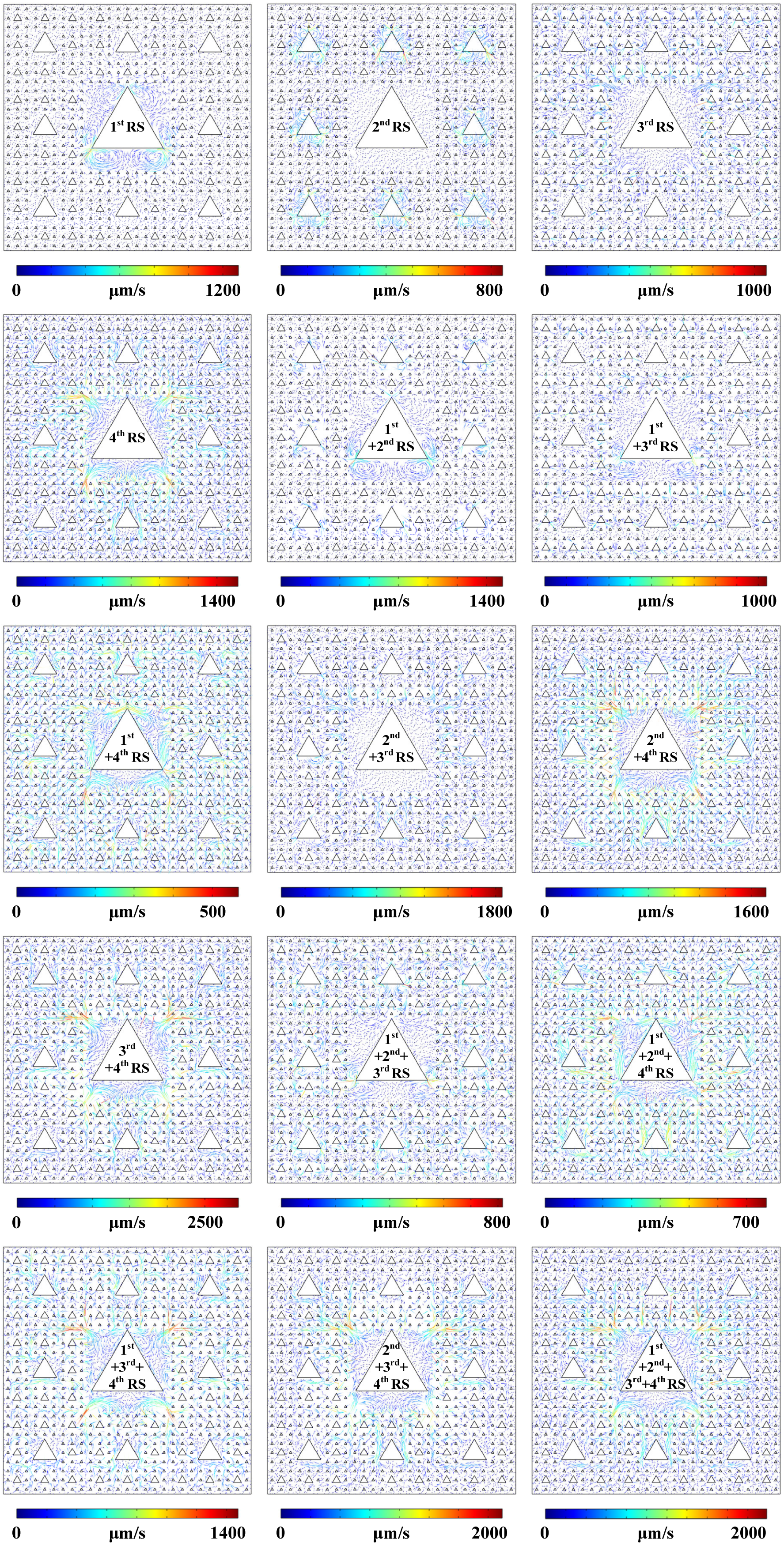
1st RS
2nd RS
3rd RS
0 µm/s 1200
0 µm/s 800
0 µm/s 1000
4th RS
1st +2nd RS
1st +3rd RS
0 µm/s 1400
0 µm/s 1400
0 µm/s 1000
1st +4th RS
2nd +3rd RS
2nd +4th RS
0 µm/s 500
0 µm/s 1800
0 µm/s 1600
3rd +4th RS
1st +2nd+ 3rd RS
1st +2nd+ 4th RS
0 µm/s 2500
0 µm/s 800
0 µm/s 700
1st +3rd+ 4th RS
2nd +3rd+ 4th RS
1st +2nd+ 3rd+4th RS
0 µm/s 1400
0 µm/s 2000
0 µm/s 2000

(b)

Figure S4. (Color online). Particle trajectories generated in the 4-stage pseudo-Sierpiński-carpet shaped chamber with different cross-sections under the excitation of different-stage radiation surfaces. **(a)** Circular cross-section. **(b)** Triangular cross-section.

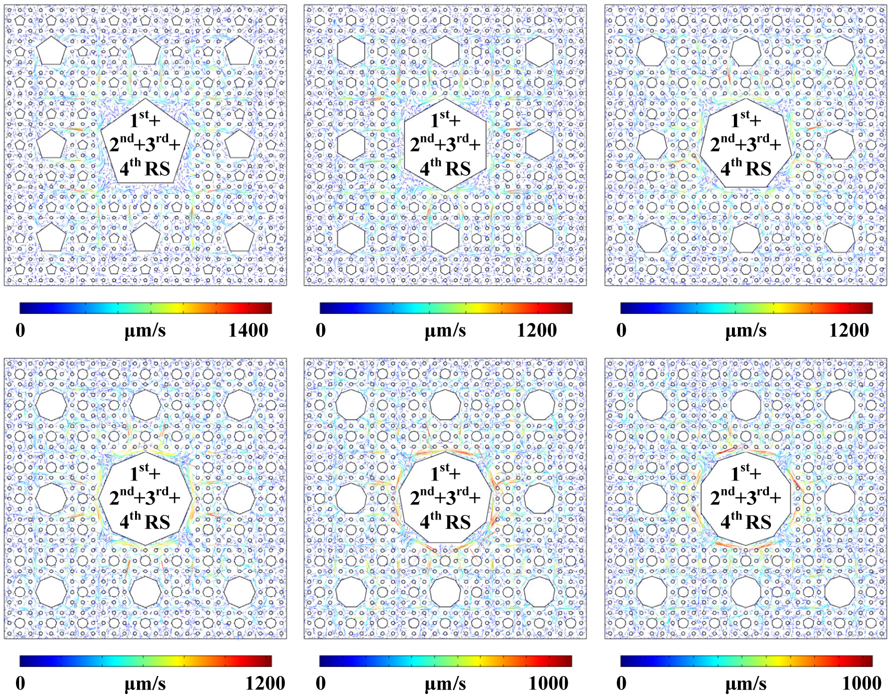

Figure S5. (Color online). Particle trajectories generated in the 4-stage pseudo-Sierpiński-carpet shaped chamber with different regular polygonal cross-sections under the excitation of 1st+2nd+3rd+4th RS.

5. Acoustofluidic patterns and particle trajectories generated in the 3- and 4-stage pesudo-Sierpiński-carpet shaped chambers with other cross-sections

More simulated results of the acoustofluidic fields and particle motion trajectories generated within 3- and 4-stage pseudo-Sierpiński-carpet-shaped chambers featuring alternative cross-sectional configurations (namely, Reuleaux polygon, pentagram and concave square) are provided in the subsequent figures as supporting information. Given that the underlying mechanisms and evolutionary principles governing the diversified acoustofluidic distributions have been thoroughly elucidated in the main text, no further detailed explanations are included in the supplementary material.

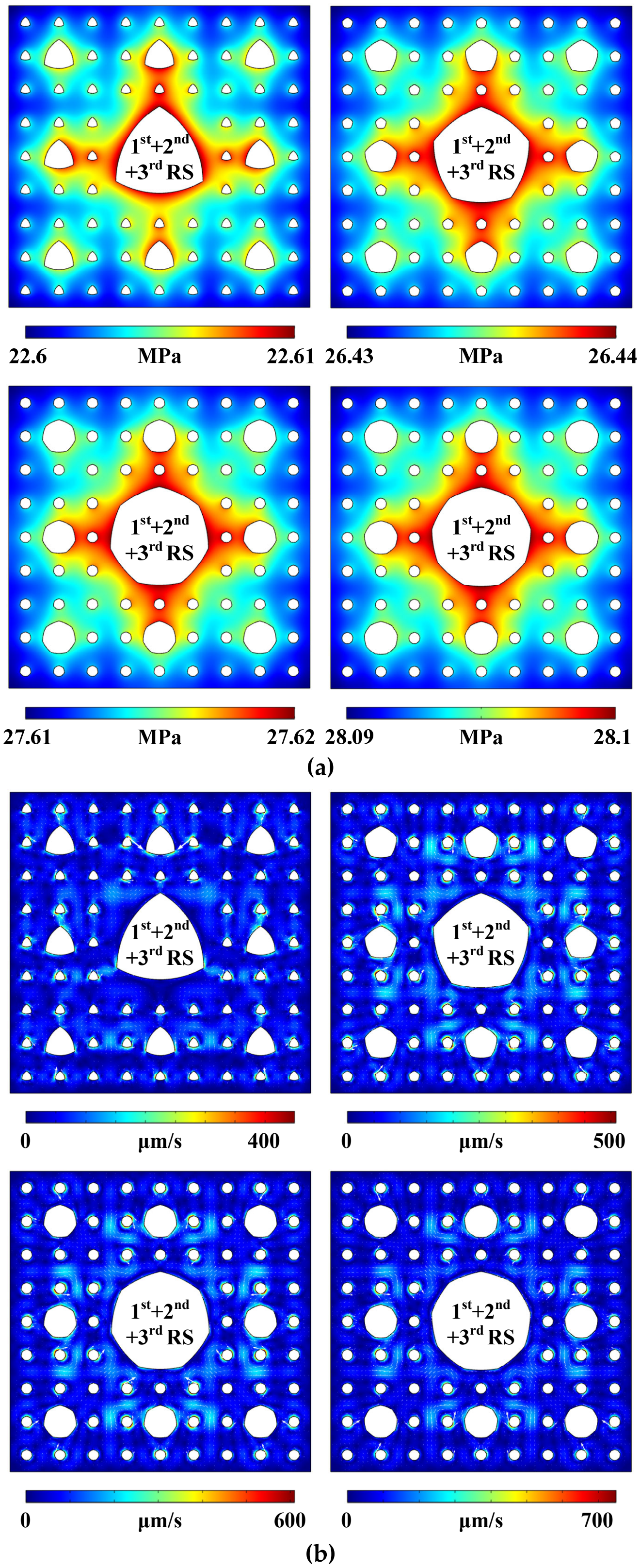
1st+2nd +3rd RS
22.6
MPa
22.61
26.43
MPa
26.44
27.61
MPa
27.62
28.09
MPa
28.1
(a)
0
μm/s
400
0
μm/s
500
0
μm/s
600
0
μm/s
700
(b)

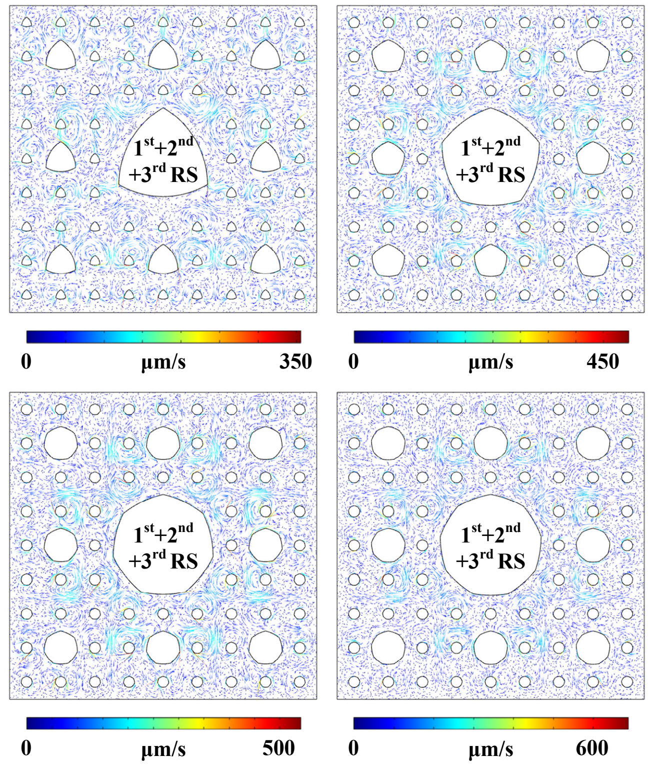

(c)

Figure S6. (Color online). Acoustofluidic fields and particle trajectories generated in the 3-stage pseudo-Sierpiński-carpet shaped chamber with different Reuleaux polygonal cross-sections under the excitation of 1st+2nd+3rd RS. **(a)** Pattern of sound pressure field. **(b)** Pattern of acoustic streaming field. **(c)** Pattern of micro particle trajectory at a given time (10 s).

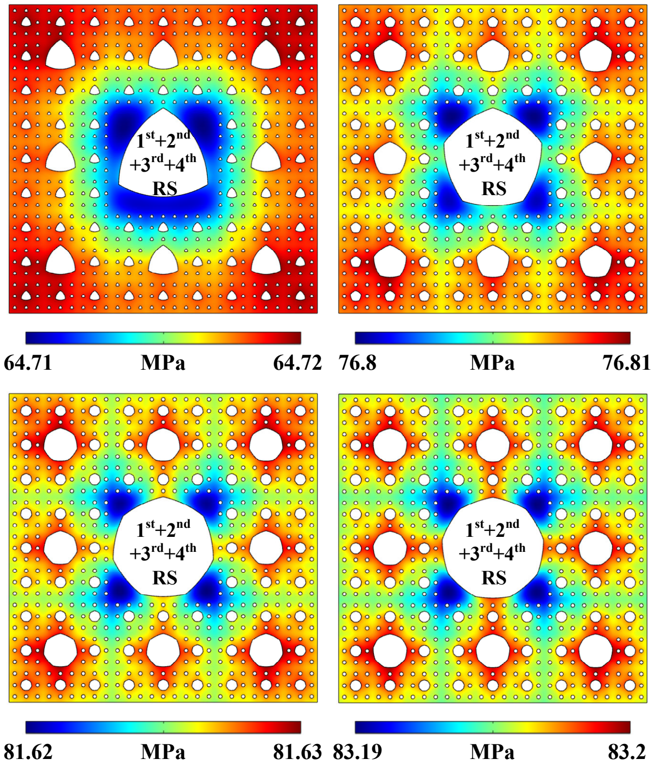

(a)

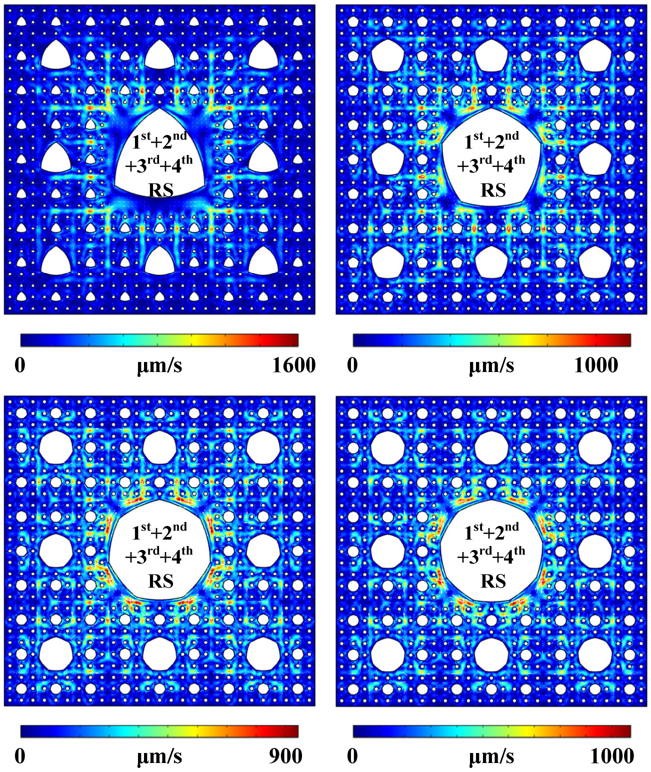

(b)

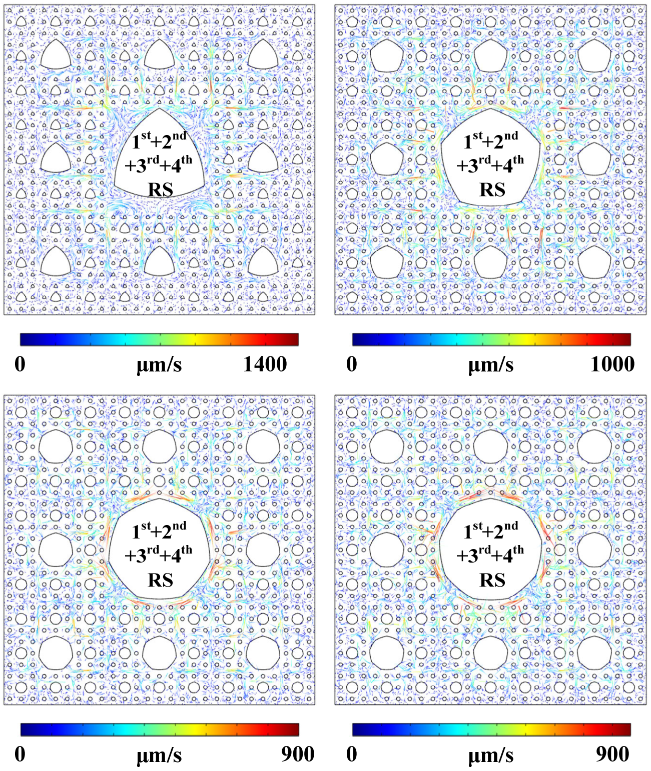

(c)

Figure S7. (Color online). Acoustofluidic fields and particle trajectories generated in the 4-stage pseudo-Sierpiński-carpet shaped chamber with different Reuleaux polygonal cross-sections under the excitation of $1^{st}+2^{nd}+3^{rd}+4^{th}$ RS. **(a)** Pattern of sound pressure field. **(b)** Pattern of acoustic streaming field. **(c)** Pattern of micro particle trajectory at a given time (10 s).

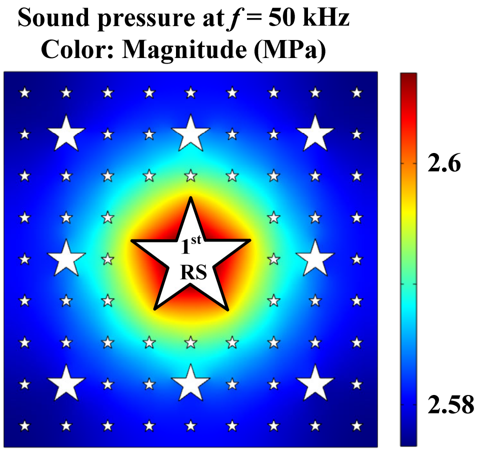

(a)

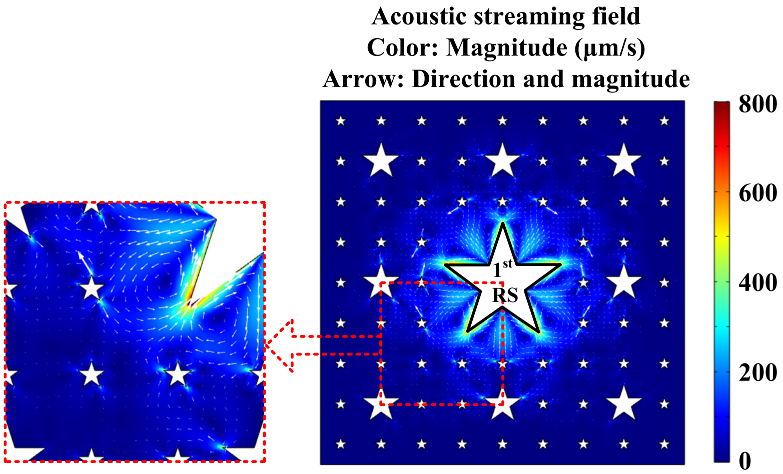

(b)

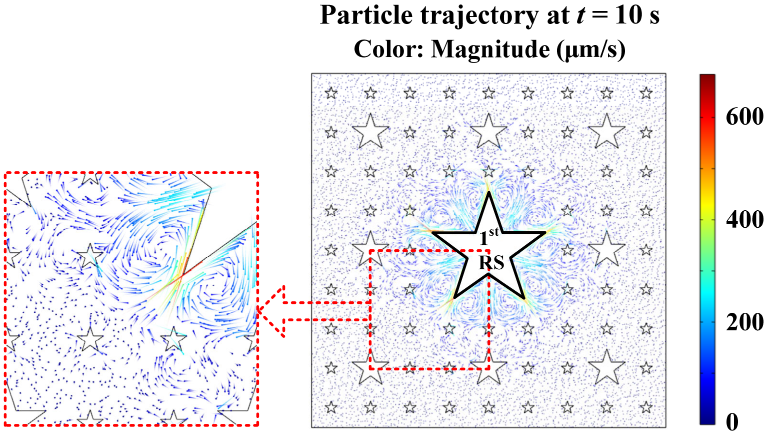

(c)

Figure S8. (Color online). Acoustofluidic fields and particle trajectories generated in the 3-stage pseudo-Sierpiński-carpet shaped chamber with pentagramal cross-section under the excitation of 1st RS. **(a)** Pattern of sound pressure field. **(b)** Pattern of acoustic streaming field. **(c)** Pattern of micro particle trajectory at a given time (10 s).

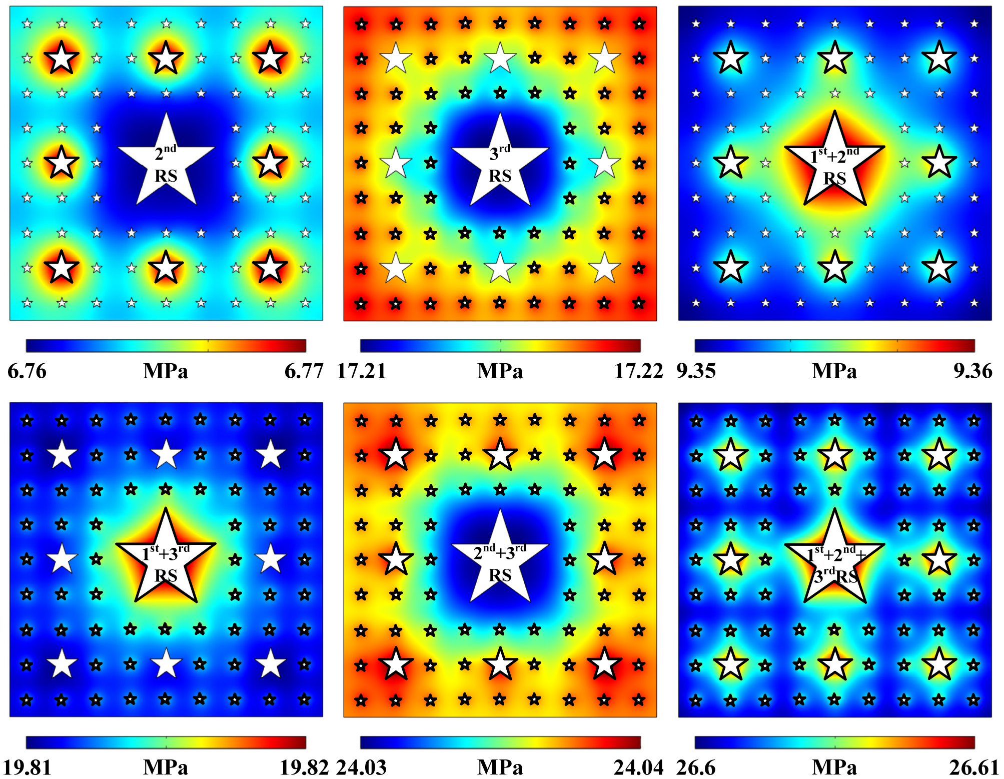
2nd RS
3rd RS
1st+2nd RS
6.76 MPa 6.77
17.21 MPa 17.22
9.35 MPa 9.36
1st+3rd RS
2nd+3rd RS
1st+2nd+3rd RS
19.81 MPa 19.82
24.03 MPa 24.04
26.6 MPa 26.61

(a)

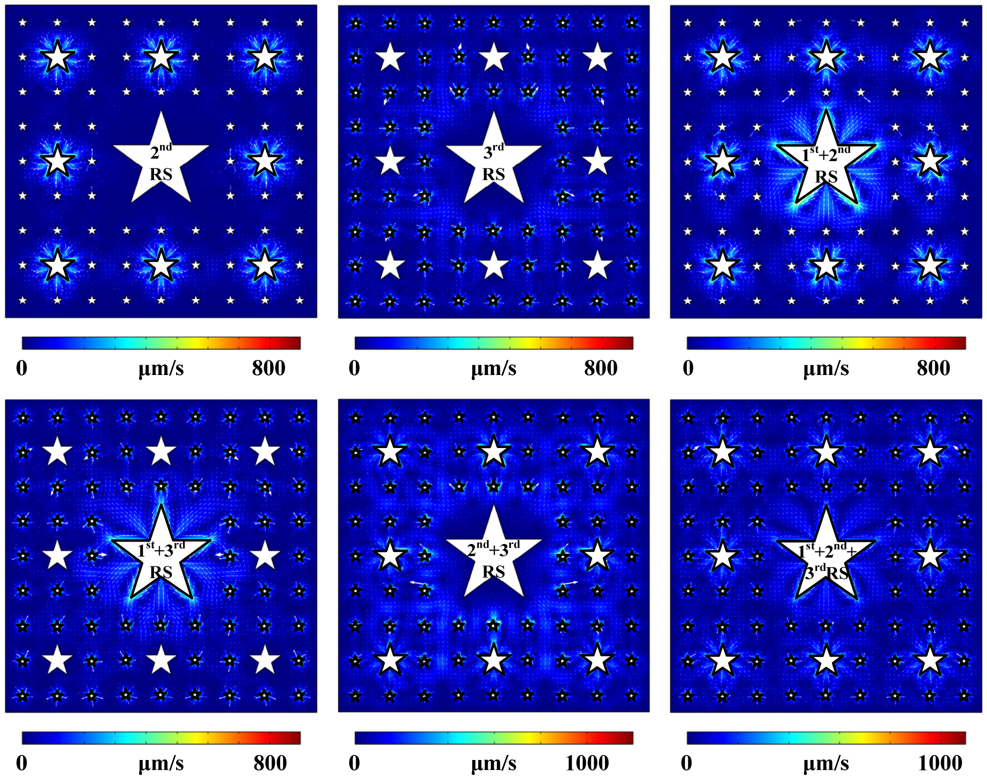
2nd RS
3rd RS
1st+2nd RS
0 μm/s 800
0 μm/s 800
0 μm/s 800
1st+3rd RS
2nd+3rd RS
1st+2nd+3rd RS
0 μm/s 800
0 μm/s 1000
0 μm/s 1000

(b)

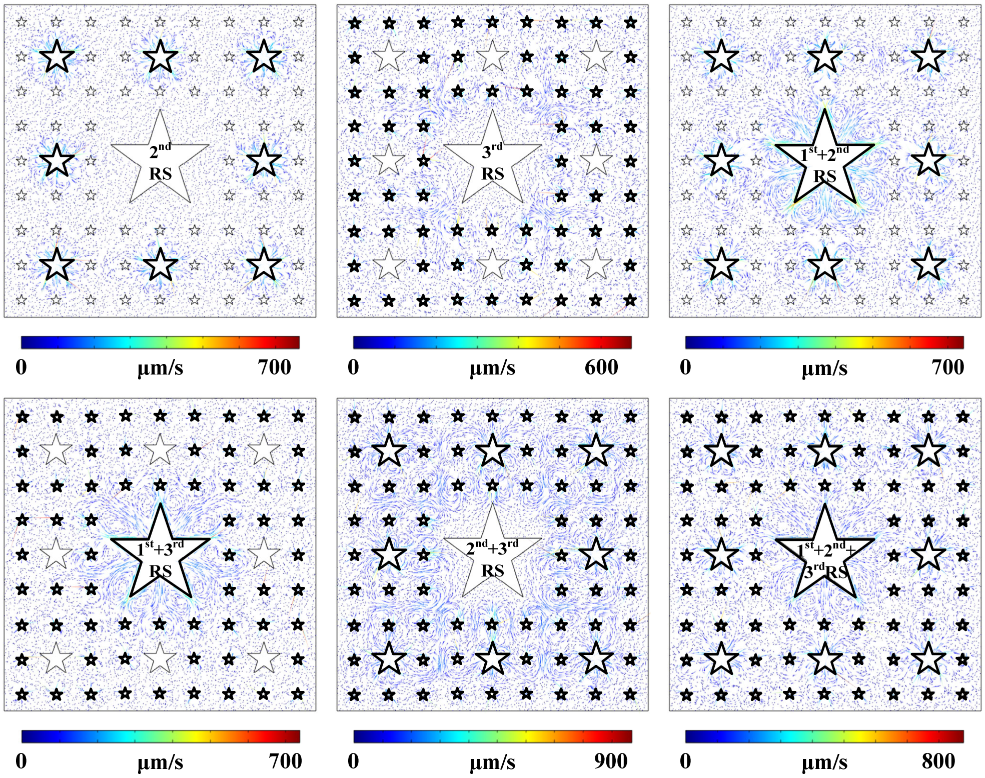

(c)

Figure S9. (Color online). Acoustofluidic fields and particle trajectories generated in the 3-stage pseudo-Sierpiński-carpet shaped chamber with pentagramal cross-section under the excitation of different-stage radiation surfaces. **(a)** Pattern of sound pressure field. **(b)** Pattern of acoustic streaming field. **(c)** Pattern of micro particle trajectory at a given time (10 s).

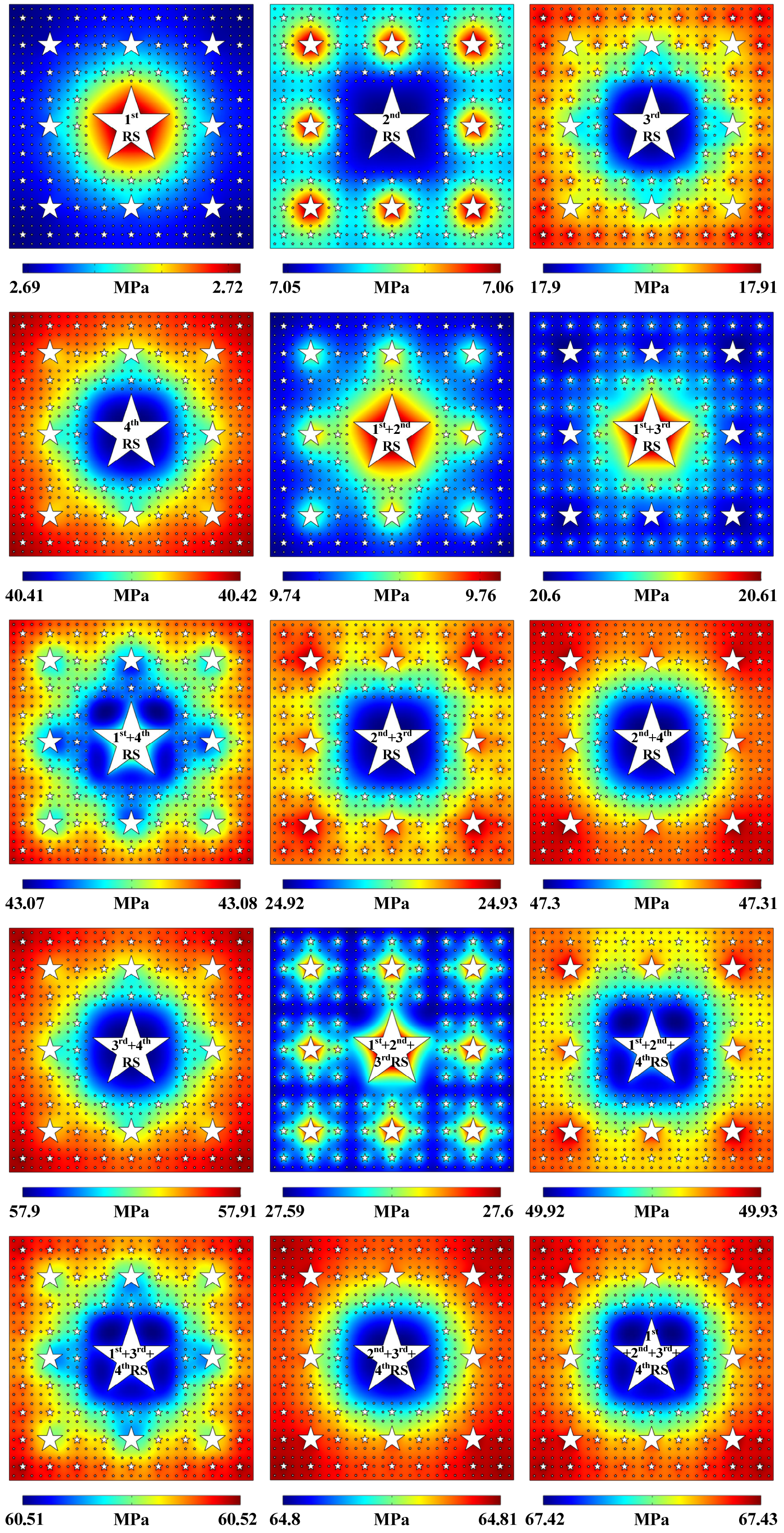

1st RS
2.69 MPa 2.72
2nd RS
7.05 MPa 7.06
3rd RS
17.9 MPa 17.91
4th RS
40.41 MPa 40.42
1st+2nd RS
9.74 MPa 9.76
1st+3rd RS
20.6 MPa 20.61
1st+4th RS
43.07 MPa 43.08
2nd+3rd RS
24.92 MPa 24.93
2nd+4th RS
47.3 MPa 47.31
3rd+4th RS
57.9 MPa 57.91
1st+2nd+3rd RS
27.59 MPa 27.6
1st+2nd+4th RS
49.92 MPa 49.93
1st+3rd+4th RS
60.51 MPa 60.52
2nd+3rd+4th RS
64.8 MPa 64.81
1st+2nd+3rd+4th RS
67.42 MPa 67.43

(a) 125

126

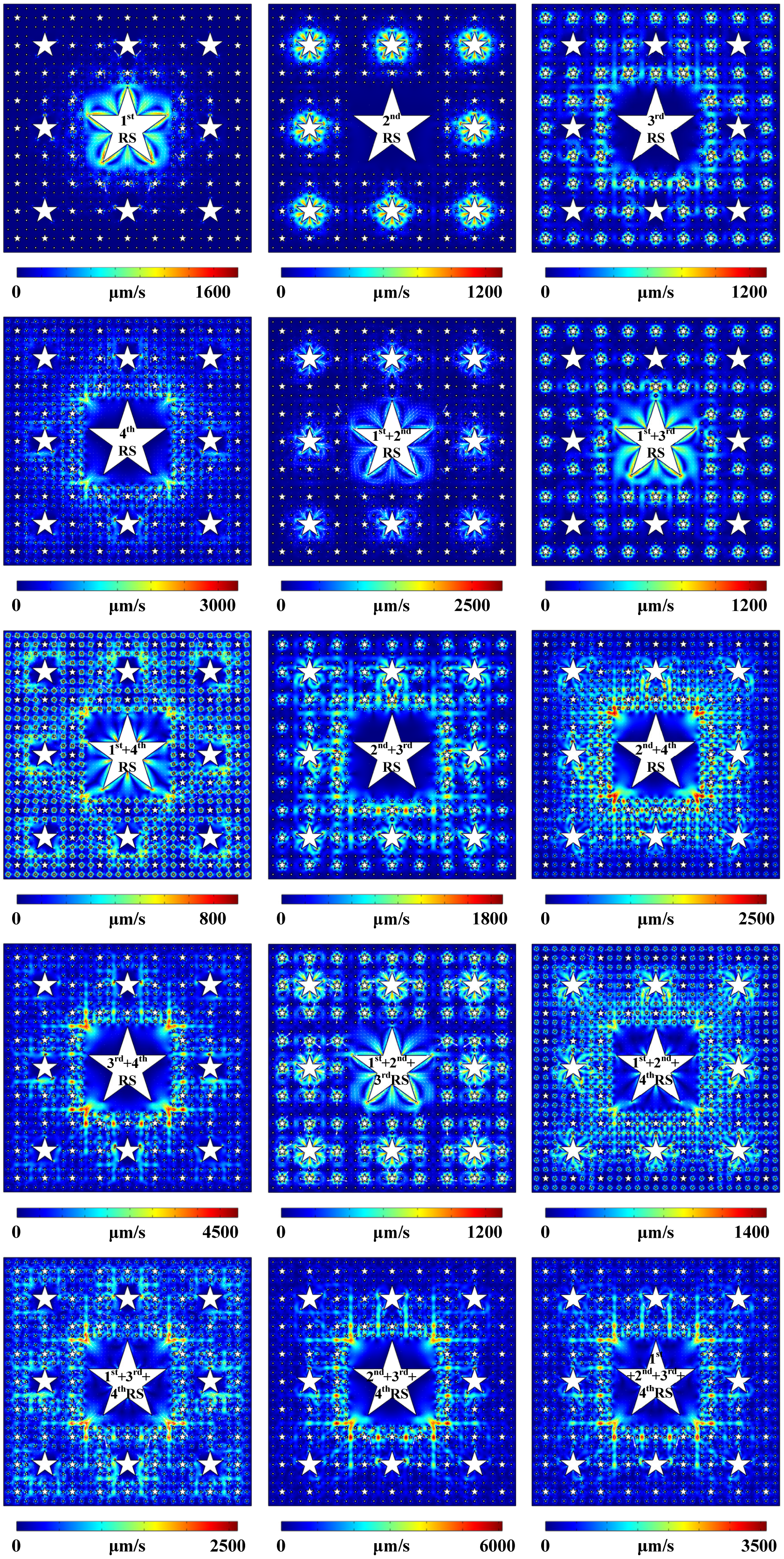
1st RS
2nd RS
3rd RS
0 µm/s 1600
0 µm/s 1200
0 µm/s 1200
4th RS
1st+2nd RS
1st+3rd RS
0 µm/s 3000
0 µm/s 2500
0 µm/s 1200
1st+4th RS
2nd+3rd RS
2nd+4th RS
0 µm/s 800
0 µm/s 1800
0 µm/s 2500
3rd+4th RS
1st+2nd+3rd RS
1st+2nd+4th RS
0 µm/s 4500
0 µm/s 1200
0 µm/s 1400
1st+3rd+4th RS
2nd+3rd+4th RS
1st+2nd+3rd+4th RS
0 µm/s 2500
0 µm/s 6000
0 µm/s 3500

(b)

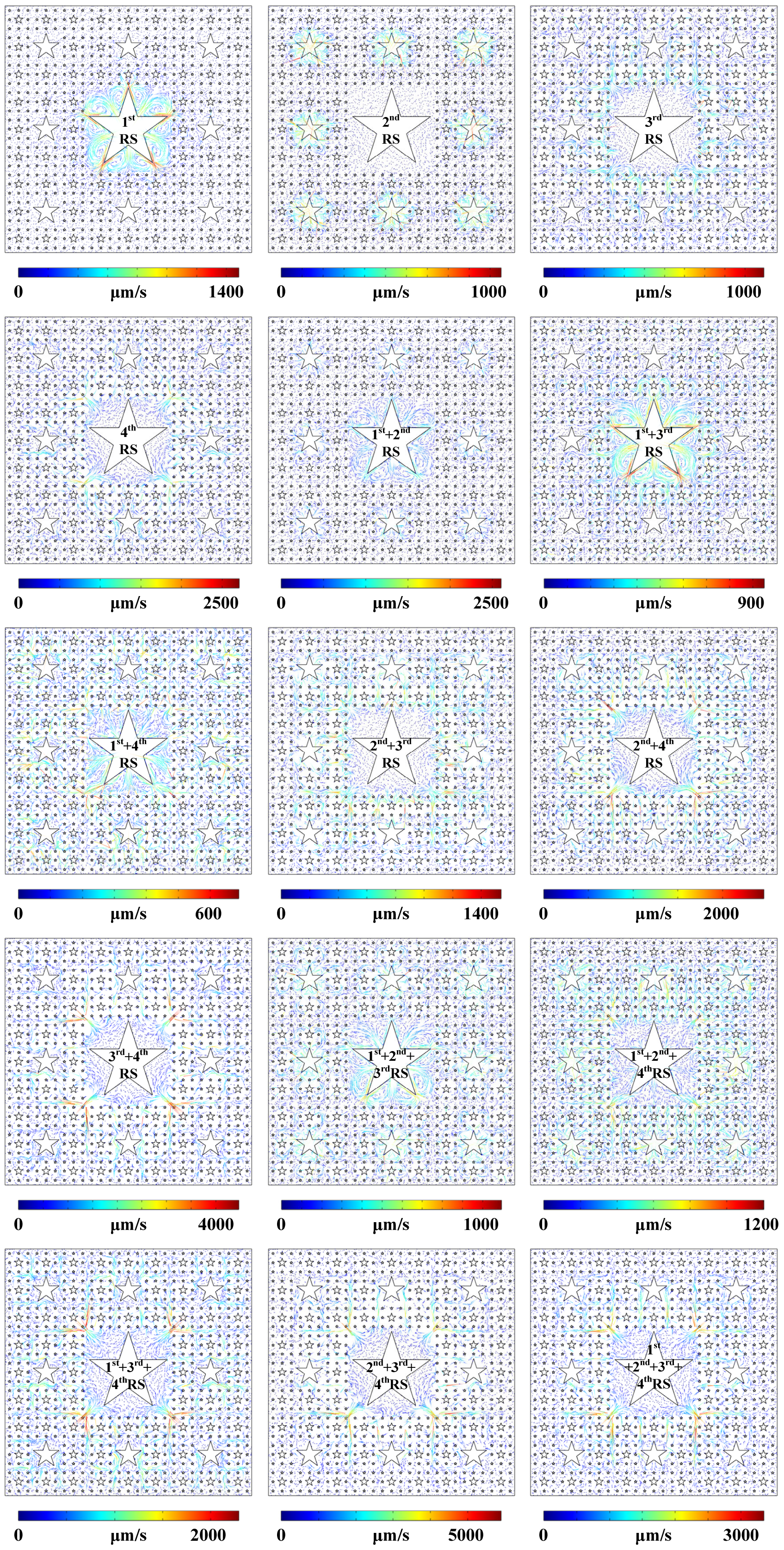

(c)

Figure S10. (Color online). Acoustofluidic fields and particle trajectories generated in the 4-stage pseudo-Sierpiński-carpet shaped chamber with pentagramal cross-section under

the excitation of different-stage radiation surfaces. **(a)** Pattern of sound pressure field. **(b)** Pattern of acoustic streaming field. **(c)** Pattern of micro particle trajectory at a given time (10 s).

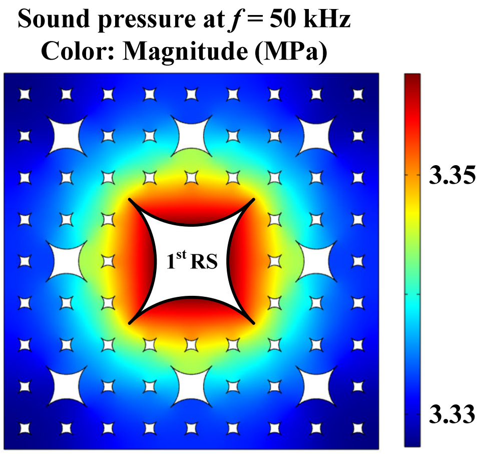

(a)

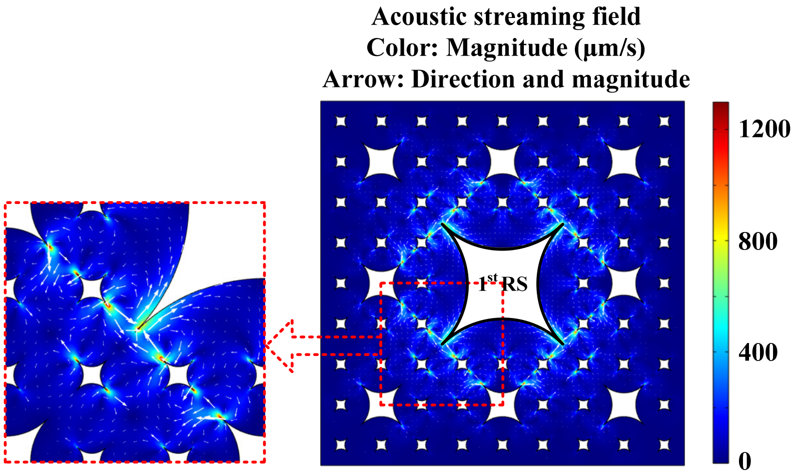

(b)

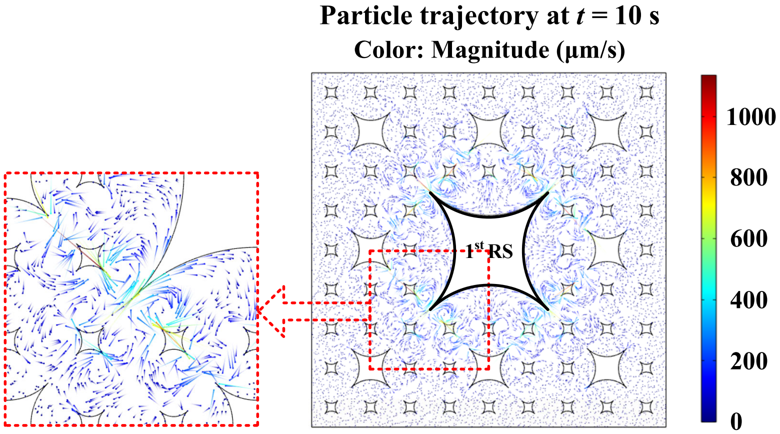

(c)

Figure S11. (Color online). Acoustofluidic fields and particle trajectories generated in the 3-stage pseudo-Sierpiński-carpet shaped chamber with concave square cross-section under the excitation of 1st RS. **(a)** Pattern of sound pressure field. **(b)** Pattern of acoustic streaming field. **(c)** Pattern of micro particle trajectory at a given time (10 s).

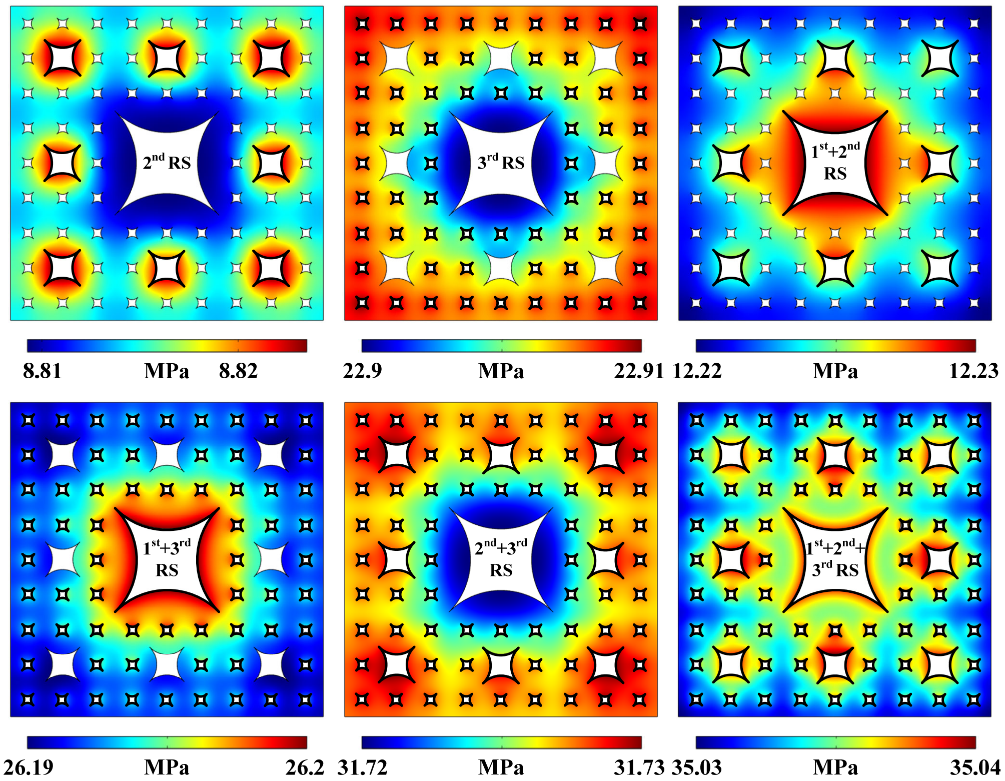
2nd RS
3rd RS
1st+2nd RS
8.81 MPa 8.82
22.9 MPa 22.91
12.22 MPa 12.23
1st+3rd RS
2nd+3rd RS
1st+2nd+3rd RS
26.19 MPa 26.2
31.72 MPa 31.73
35.03 MPa 35.04

(a)

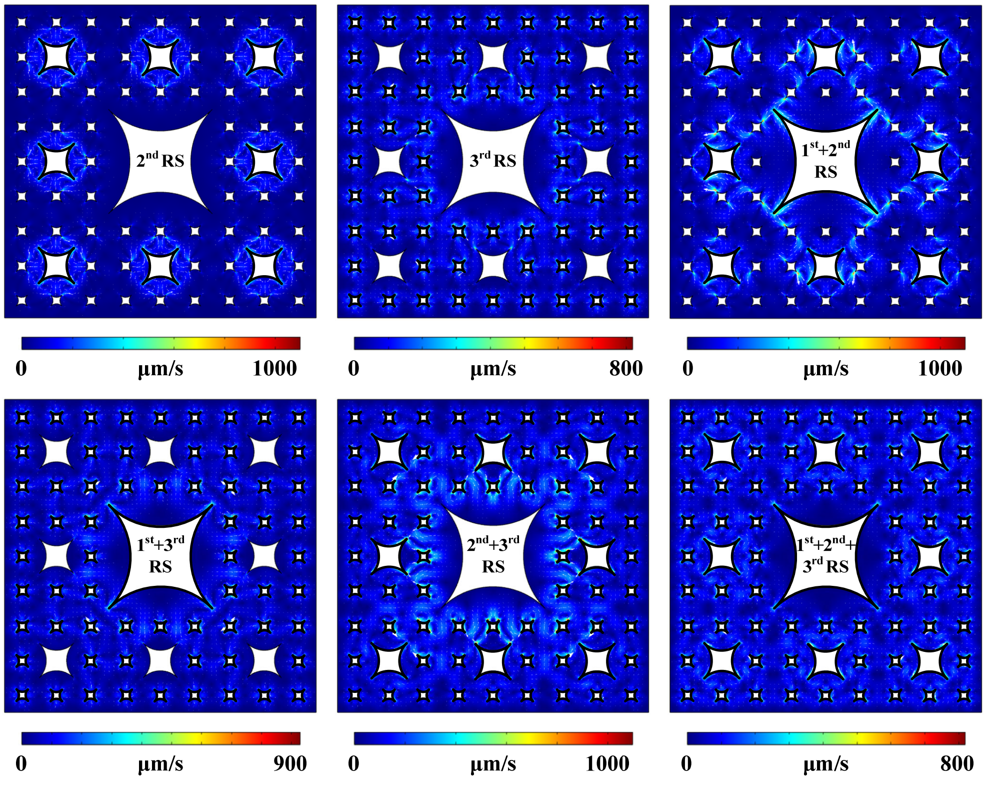
2nd RS
3rd RS
1st+2nd RS
0 μm/s 1000
0 μm/s 800
0 μm/s 1000
1st+3rd RS
2nd+3rd RS
1st+2nd+3rd RS
0 μm/s 900
0 μm/s 1000
0 μm/s 800

(b)

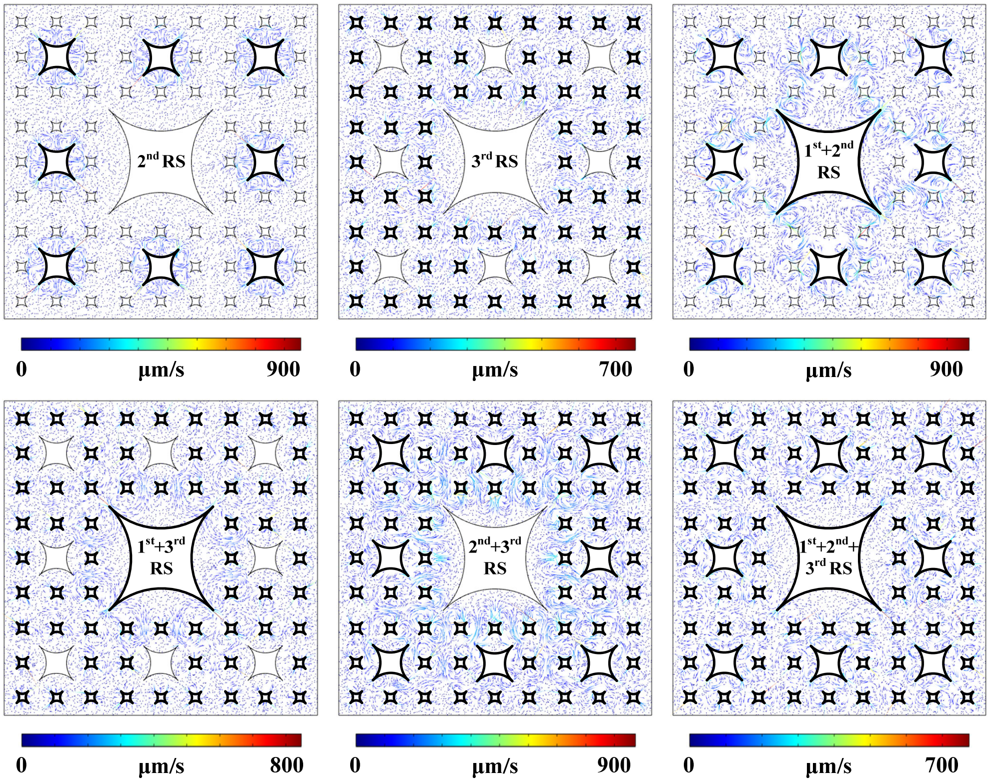

(c)

Figure S12. (Color online). Acoustofluidic fields and particle trajectories generated in the 3-stage pseudo-Sierpiński-carpet shaped chamber with concave square cross-section under the excitation of different-stage radiation surfaces. **(a)** Pattern of sound pressure field. **(b)** Pattern of acoustic streaming field. **(c)** Pattern of micro particle trajectory at a given time (10 s).

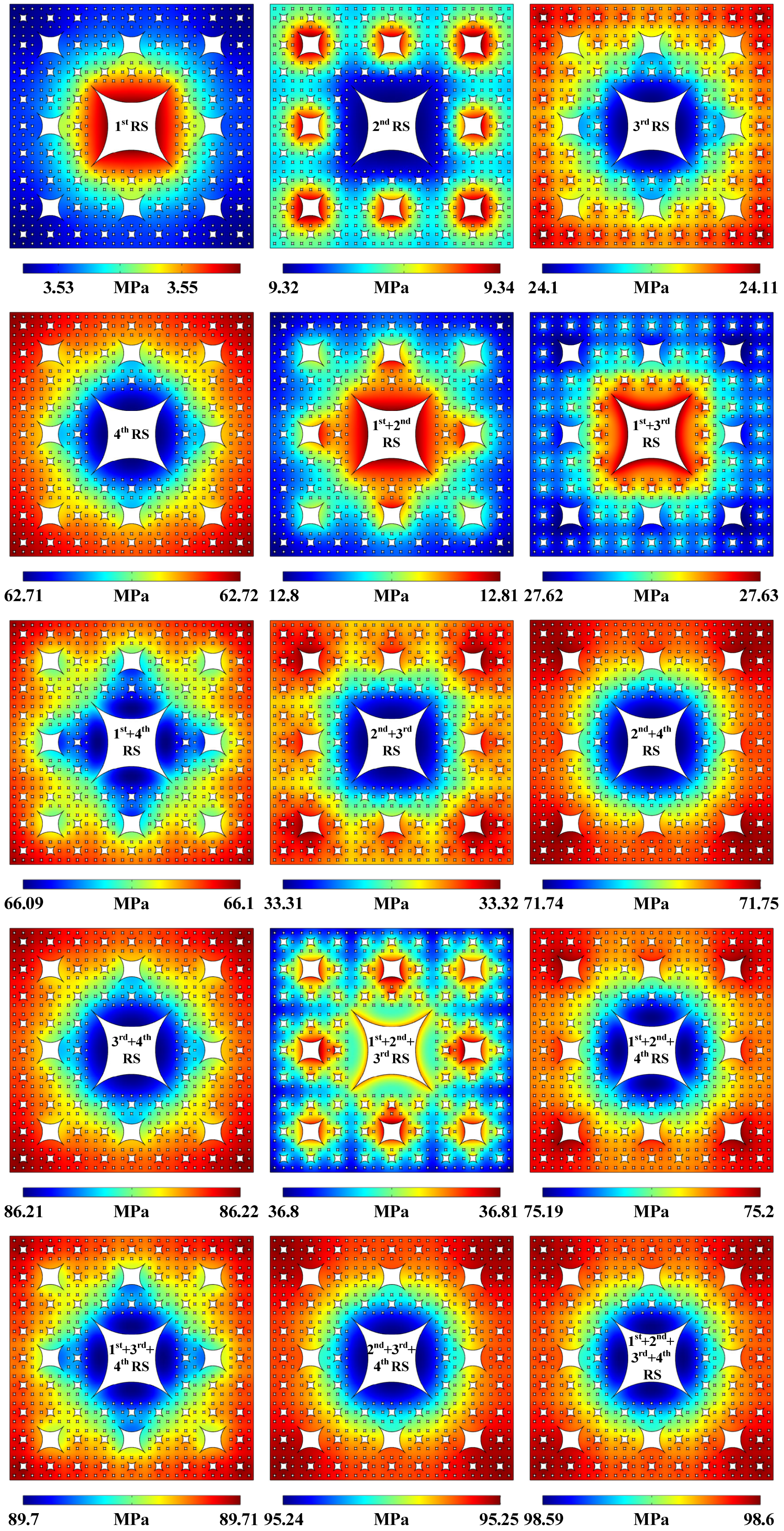
1st RS
2nd RS
3rd RS
3.53 MPa 3.55
9.32 MPa 9.34
24.1 MPa 24.11
4th RS
1st+2nd RS
1st+3rd RS
62.71 MPa 62.72
12.8 MPa 12.81
27.62 MPa 27.63
1st+4th RS
2nd+3rd RS
2nd+4th RS
66.09 MPa 66.1
33.31 MPa 33.32
71.74 MPa 71.75
3rd+4th RS
1st+2nd+ 3rd RS
1st+2nd+ 4th RS
86.21 MPa 86.22
36.8 MPa 36.81
75.19 MPa 75.2
1st+3rd+ 4th RS
2nd+3rd+ 4th RS
1st+2nd+ 3rd+4th RS
89.7 MPa 89.71
95.24 MPa 95.25
98.59 MPa 98.6

(a)

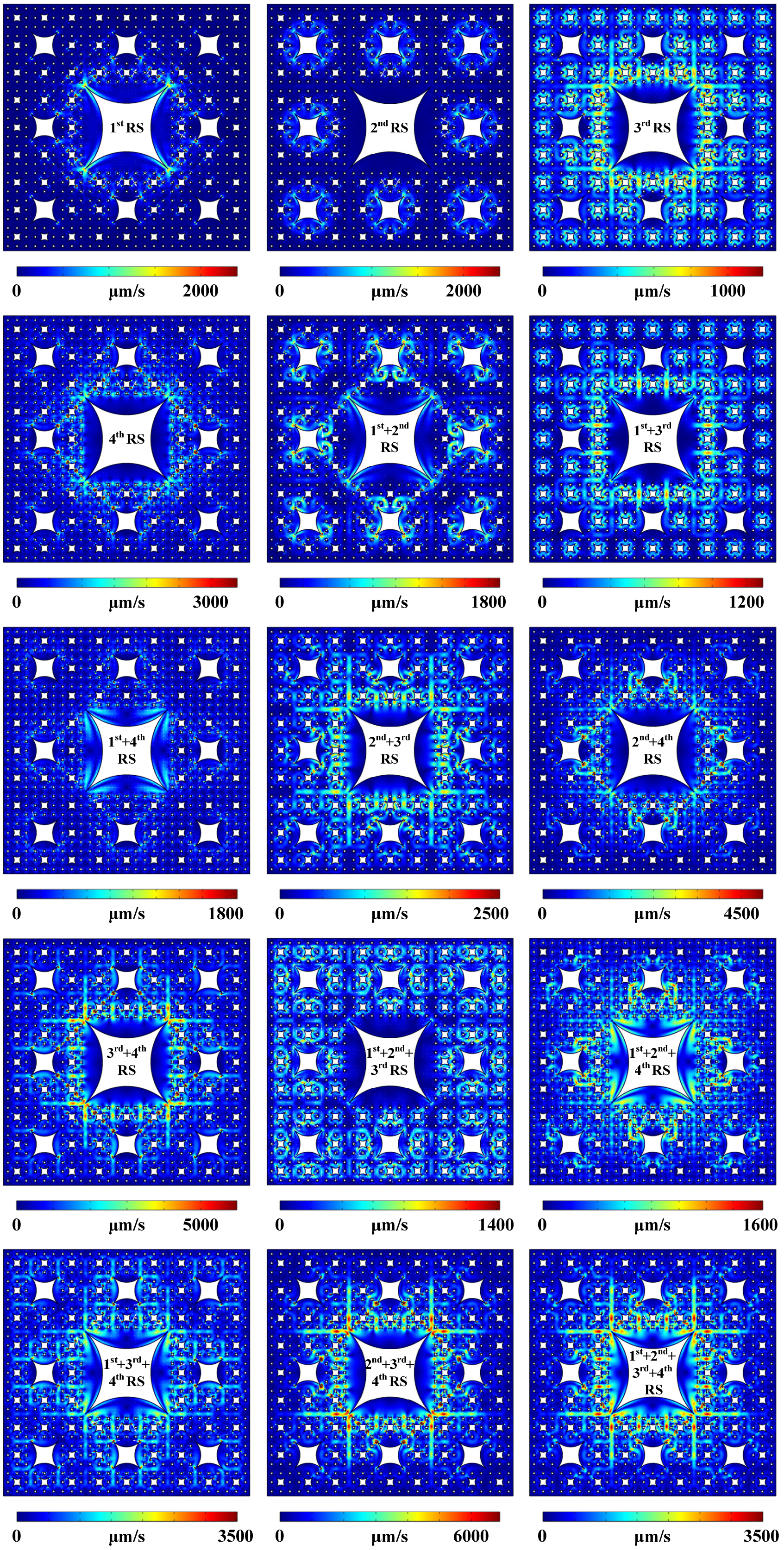
1st RS
0 μm/s 2000
2nd RS
0 μm/s 2000
3rd RS
0 μm/s 1000
4th RS
0 μm/s 3000
1st+2nd RS
0 μm/s 1800
1st+3rd RS
0 μm/s 1200
1st+4th RS
0 μm/s 1800
2nd+3rd RS
0 μm/s 2500
2nd+4th RS
0 μm/s 4500
3rd+4th RS
0 μm/s 5000
1st+2nd+ 3rd RS
0 μm/s 1400
1st+2nd+ 4th RS
0 μm/s 1600
1st+3rd+ 4th RS
0 μm/s 3500
2nd+3rd+ 4th RS
0 μm/s 6000
1st+2nd+ 3rd+4th RS
0 μm/s 3500

(b)

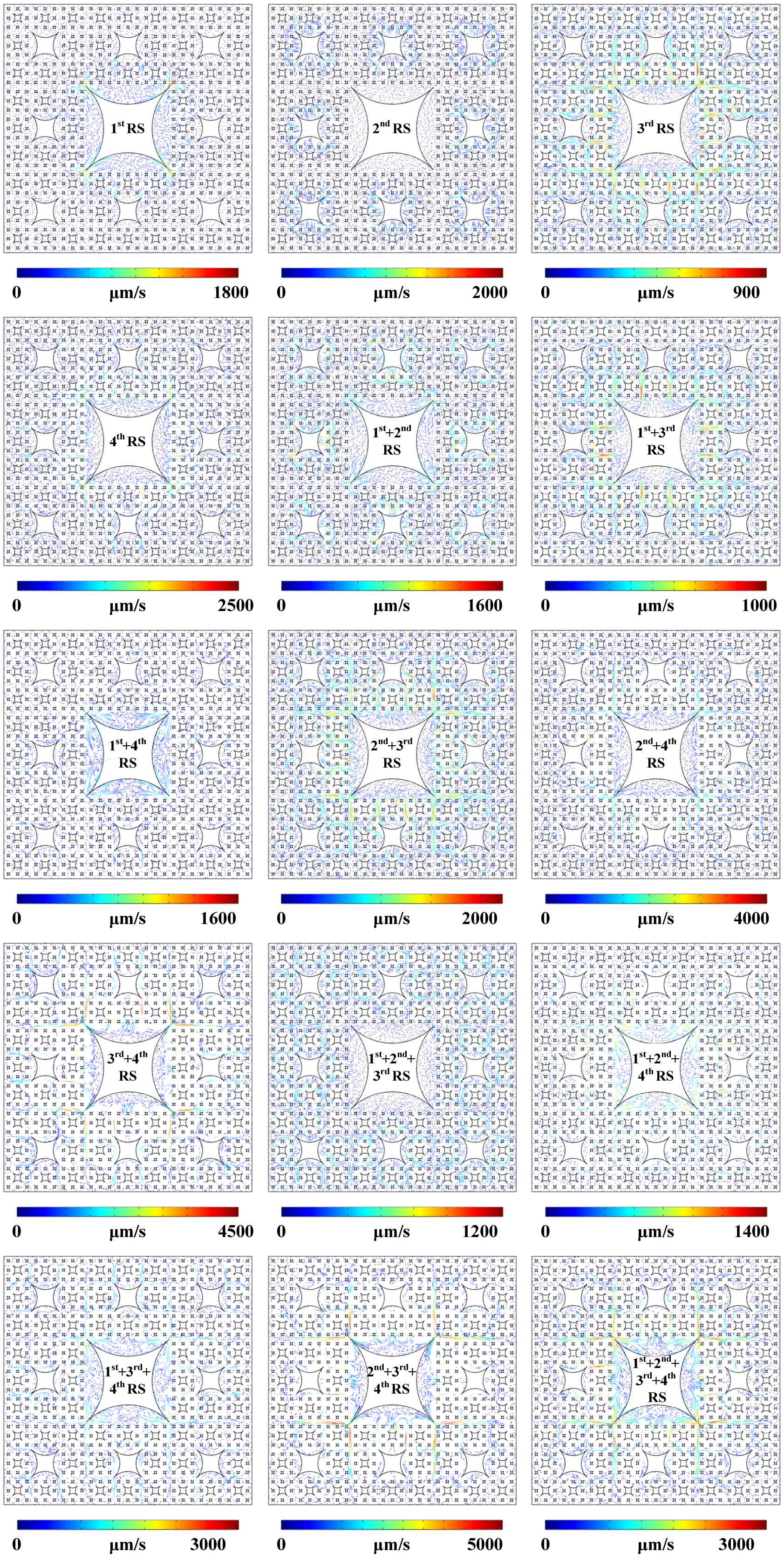
1st RS
0 µm/s 1800
2nd RS
0 µm/s 2000
3rd RS
0 µm/s 900
4th RS
0 µm/s 2500
1st+2nd RS
0 µm/s 1600
1st+3rd RS
0 µm/s 1000
1st+4th RS
0 µm/s 1600
2nd+3rd RS
0 µm/s 2000
2nd+4th RS
0 µm/s 4000
3rd+4th RS
0 µm/s 4500
1st+2nd+3rd RS
0 µm/s 1200
1st+2nd+4th RS
0 µm/s 1400
1st+3rd+4th RS
0 µm/s 3000
2nd+3rd+4th RS
0 µm/s 5000
1st+2nd+3rd+4th RS
0 µm/s 3000

(c)

Figure S13. (Color online). Acoustofluidic fields and particle trajectories generated in the 4-stage pseudo-Sierpiński-carpet shaped chamber with concave square cross-section under the excitation of different-stage radiation surfaces. **(a)** Pattern of sound pressure field. **(b)** Pattern of acoustic streaming field. **(c)** Pattern of micro particle trajectory at a given time (10 s).

Table S1. Averaged acoustic streaming velocity magnitude (μm/s) under all circumstances.

cross-section	RS and stage	1st	2nd	3rd	4th	1st+2nd	1st+3rd	1st+4th	2nd+3rd	2nd+4th	3rd+4th	1st+2nd+3rd	1st+2nd+4th	1st+3rd+4th	2nd+3rd+4th	1st+2nd+3rd+4th
circle	3-stage	2.72	4.66	16.2	N/A	44.5	54.6	N/A	65.2	N/A	N/A	85.2	N/A	N/A	N/A	N/A
	4-stage	4.55	6.29	23.1	63.9	42.5	119	52.2	146	135	194	178	95.4	113	266	166
triangle	3-stage	15.1	28.2	43.1	N/A	38.5	44	N/A	69.4	N/A	N/A	56.3	N/A	N/A	N/A	N/A
	4-stage	55.9	57.8	106	242	88.1	96.7	137	172	307	460	120	174	293	508	315
pentagram	3-stage	36.6	37.7	46	N/A	63	51.5	N/A	89.4	N/A	N/A	73.5	N/A	N/A	N/A	N/A
	4-stage	93.2	100	159	351	175	150	188	290	458	691	211	262	438	750	477
concave square	3-stage	62.8	49.8	62.8	N/A	80.7	64.8	N/A	108	N/A	N/A	79.5	N/A	N/A	N/A	N/A
	4-stage	109	134	216	348	208	180	170	382	543	849	217	266	510	955	562

regular polygon		pentagon	hexagon	heptagon	octagon	nonagon	decagon
RS and stage	1st+2nd+3rd 3-stage	57.2	61.4	65.9	68.1	71.3	73.5
	1st+2nd+3rd+4th 4-stage	221	210	227	218	211	197

Reuleaux polygon		Reuleaux triangle	Reuleaux pentagon	Reuleaux heptagon	Reuleaux nonagon
RS and stage	1st+2nd+3rd 3-stage	50.6	65.9	73.5	77
	1st+2nd+3rd+4th 4-stage	202	183	174	186

References

1. Fu, Y.Q.; Luo, J.K.; Nguyen, N.T.; Walton, A.J.; Flewitt. A.J.; Zu, X.T.; Li, B.; McHale, G.; Matthews, A.L.; Iborra, E.; Du, H.; Milne, W.I. Advances in piezoelectric thin films for acoustic biosensors, acoustofluidics and lab-on-chip applications. *Prog. Mater. Sci.* **2017**, *89*, 31-91.
2. Tang, Q.; Zhou, S.; Huang, L.; Chen, Z. Diversity of 2D acoustofluidic fields in an ultrasonic cavity generated by multiple vibration sources. *Micromachines* **2019**, *10*, 803.
3. Tang, Q.; Yang, Y.; Liu, P.; Wang, T.; Wang, X. Sophisticated acoustofluidic patterns generated in quasi-Sierpiński-carpet shaped chambers with heterogeneous radiation surface distributions. *Phys. Scr.* **2022**, *97*, 085209.

Disclaimer/Publisher's Note: The statements, opinions and data contained in all publications are solely those of the individual author(s) and contributor(s) and not of MDPI and/or the editor(s). MDPI and/or the editor(s) disclaim responsibility for any injury to people or property resulting from any ideas, methods, instructions or products referred to in the content.
